# Emerging demand-side flexible resources accelerate China’s power system transition toward carbon neutrality

**DOI:** 10.1016/j.isci.2025.112372

**Published:** 2025-04-08

**Authors:** Hongyi Wei, Ning Zhang, Ershun Du, Haiyang Jiang, Zhenyu Zhuo, Michael R. Davidson, Weiran Li, Peng Wang, Jinyu Xiao, Chongqing Kang

**Affiliations:** 1 Tsinghua University, Department of Electrical Engineering, Beijing 100084, China; 2 Tsinghua University, Institute of Climate Change and Sustainable Development, Beijing 100084, China; 3 Beijing Sankuai Online Technology Co., Ltd, Beijing 100102, China; 4 University of California, San Diego, Department of Mechanical and Aerospace Engineering, La Jolla, CA 92039, USA; 5 Global Energy Interconnection Development and Cooperation Organization, Beijing 100031, China

**Keywords:** Energy engineering, Energy systems, Energy management

## Abstract

This study explores how demand-side flexible resources (DSFR) contribute to China’s power system transition toward carbon neutrality. We find that approximately 20% of the costs associated with the carbon neutrality transition would be reduced by incorporating DSFR accounting for one-fourth of peak load capacity. Such reduction mainly comes from substituting costly energy storage and flexible generation units with diversified low-carbon demand resources and reducing the lock-in of thermal units in the medium term. We find that flexible electric vehicle charging and power-to-hydrogen load contribute the most to flexible load demands among DSFR, assisting regional power balancing and renewable energy accommodation. Controllable power reserve capacity of demand response resources benefits less operating expensive power reserve resources, reducing the need for gas generation by 42% in 2060. We further find load demand potentials show great influences on transition costs and system morphology development, most notably flexible power-to-hydrogen load demands.

## Highlights

• Emerging DSFR leads to considerable carbon neutrality cost reductions
• Various DSFRs have synergistic effects on low-carbon capacity structure transformation
• Cost-effective flexible loads and power reserves from DSFR support system operation

## Graphical abstract

**Figure:**
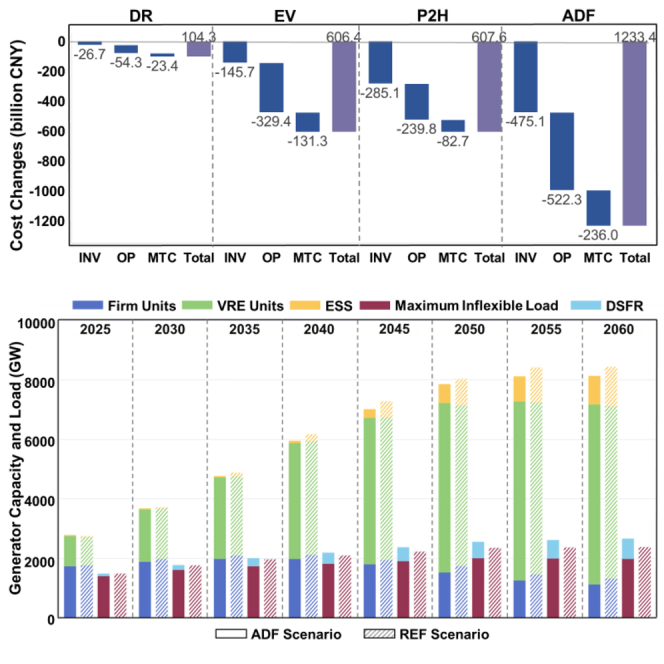

## Introduction

China has announced targets to achieve carbon peaking and carbon neutrality by 2030 and 2060, respectively, for which a high proportion of variable renewable energy (VRE) generation will be essential.^1^^,^^2^^,^^3^^,^^4^ Wind and solar photovoltaic generation accounted for 13.8% of national electricity consumption in 2022 and must grow to over 65% by 2060.^5^ Accommodating high VRE penetrations requires greater grid flexibility, which can be met from several sources^6^^,^^7^: non-VRE generator flexibility,^8^ grid expansion and flexible operations,^9^^,^^10^^,^^11^ energy storage,^12^^,^^13^ and demand-side flexibility.^14^^,^^15^^,^^16^ Due to China’s large capacity of coal-fired power generation, much attention has been paid to improving costs and VRE integration through thermal power plant flexibility,^17^^,^^18^^,^^19^^,^^20^ in addition to large roles for storage, grid expansion and enhanced power system operations.^21^ Demand-side resources are identified as important potential contributors to system flexibility,^22^^,^^23^^,^^24^ though they are rarely considered systematically in decarbonization pathway modeling.

Demand-side flexible resources (DSFR) include industrial and residential demand response (DR),^25^ flexible electric vehicle (FEV) charging and discharging,^26^ and power-to-hydrogen (P2H) resources.^27^ DR resources support the integration of large-scale renewable energy resources by maintaining power load balance and providing reserves through numerous distributed controllable loads.^28^^,^^29^ Heterogeneous load resources, such as heating, ventilation, and air conditioning (HVAC) systems and small industrial loads, are aggregated to provide more flexibility for power systems. FEV includes smart charging behaviors shaping electricity load profiles and vehicle-to-grid (V2G) technology, which enables EVs to discharge stored energy to the grid, helping to maintain power balance.^30^^,^^31^ P2H involves electrolytic hydrogen production, a large electricity load that can be shaped according to power system conditions, and the conversion back to electricity (as a long-term energy storage) or for use in other sectors such as industry and transportation.^32^ These flexible resources can be managed by grid operators, load aggregators, or directly by consumers.

Demand-side resources are still in their infancy in China, though early pilots indicate the large-scale potential for key contributions to a deeply decarbonized grid. 70% of the provinces in China had introduced their DR pilot support policies by mid-2022, with a DR capacity objective of 3%–5% of the maximum system load by 2025.^33^ These policies rely on the large-scale heterogeneous responsible load dispatched by load aggregators or virtual power plants, such as the distributed HVAC systems and demand-side energy storage. The EV population in China reached 18 million in the mid of 2022, forming the largest EV stock in the world. With a predicted EV population in China growing to over 80 million in 2030 and reaching 300 million in 2060,^34^^,^^35^ policies have been announced to promote interactions between EV charging and power system operations.^36^ Fast charging stations with controlled charging and V2G capabilities are promised to be widely invested to facilitate EV load regulations. Hydrogen can be utilized as a zero-carbon fuel in a future energy system.^37^ Although China has the world’s largest hydrogen production (approx. 33 mn metric tonnes per year), the P2H industry is still small, especially in terms of green hydrogen (produced from zero-carbon electricity), with green hydrogen production projected to only reach 0.1–0.2 mn metric tonnes per year by 2025.^38^ While their development potential is relatively large, which must be considered in the power system carbon neutrality transition. Other emerging load resources, such as intelligent computing centers, could provide load regulations through load aggregators, whose response schemes remain unclear with rapidly growing loads.

Recent trends in the development of DSFR have prompted attention to their specific influences on power system operations.^39^^,^^40^^,^^41^ DSFR, and DR in particular, in seven Northern European countries was estimated to be 15–29% of the peak load, mainly from industrial, residential, and tertiary sectors.^42^ Optimal allocation of EVs and air conditioners (ACs) are analyzed to enhance renewable energy consumption for power systems, while the authors only focused on the power system transformation in Jiangsu province from 2025 to 2031, lacking a coordinated analysis with generation unit capacity expansion.^43^ Similarly, DR with fixed capacity is coordinated with other units and demands for optimal operations from 2015 to 2050 in Qinghai province.^44^ In a study of the U.S. power system with a VRE penetration of 66%, demand-side flexibility may reduce VRE curtailment from a range of 6%–9% to 2%–3% and enable better utilization of VRE units with low variable costs.^14^ Load shedding, short-term load shifting, and long-term load shifting were introduced for power system operations without considering specific operational characteristics such as EV controlled charging and V2G.^45^ The investigation of current DR programs has shown that their implementation helps to address the high variability of renewable generation and the retirement of conventional thermal generators.^15^^,^^46^ Typical demand-side technologies of VPP and V2G are examined for VRE integration and power system flexibility, which provides large load regulation potentials for power system flexibility and stability.^47^ The potential impacts of EVs were evaluated by utilizing the SWITCH-China model designed to meet carbon emission constraints within the power sector, focusing on how various EV stocks and controlled charging strategies impact power system transition.^48^ DR program implementation in long-term national energy system transition has been examined focusing on their energy demand management capability, with less consideration of power reserve capability, a significant factor supporting power system operations.^49^^,^^50^^,^^51^ However, various DSFR future potentials and roles in long-term power system transition planning have not yet been fully revealed with detailed consideration of their load-shifting and power reserving capability. Comprehensive analyses are required to examine their influences via the coordinated optimization method between power systems and these demand-side resources.

Previous studies on China’s power system to mid-century have indicated that achieving carbon neutrality may result in large transition costs and greatly influence power system morphology, such as generation mix, capacity structure, and grid infrastructure. Several studies found that VRE units would rapidly replace coal-fired units.^52^^,^^53^^,^^54^^,^^55^ Flexible resources, such as gas units, concentrated solar power (CSP) units, and energy storage system (ESS), will be required to support power system operations for carbon neutrality, resulting in large investment costs.^56^^,^^57^ A large amount of ESS capacity is required to improve overall VRE energy accommodation coordinated with wind and PV units.^58^^,^^59^^,^^60^ However, the influences of DSFR on the transition costs and power system morphology are rarely examined, especially in terms of the impacts on transition costs, generation unit installations, DSFR development pathways, and power system operations. Inflexible P2H and EV electricity demands are considered in several studies as fixed loads,^5^^,^^56^ while their deployment costs and flexible operation characteristics have not been detailedly analyzed within the power system carbon neutrality transition.

Here, we explore the roles of DSFR in the carbon neutrality transition of China’s power system, with a particular focus on the impacts on the transition cost breakdown, system morphology, demand-side flexibility distribution, and operations. We established a national coordinated generation-transmission-load-storage (GTLS) planning model with operational details incorporating DSFR to evaluate the evolution of China’s power system from 2025 to 2060 in 5-year planning increments. The results show that the utilization of DSFR contributes to reducing the additional transition cost of China’s power system toward carbon neutrality by nearly a fifth, or around 1.2 trillion CNY. Of these savings, investment, operation, and maintenance cost components account for 39%, 42%, and 19%, respectively. We found that DSFR contributes to further replacing conventional generation units with VRE units, highlighting their roles as regional power balancing resources. DSFR with load regulation capability provides power support for system operation to achieve carbon neutrality, leading to fewer investment requirements on ESS and costly flexible units such as CSP. Flexibility is mainly provided by shifting controllable loads to other periods, while the discharging behaviors of FEV and the power generation of flexible P2H resources are only utilized to satisfy the system security requirements when the power system lacks controllable power supply capacity in some critical time periods. FEV and flexible P2H resources provide the majority of shifted load demands, and controllable power reserve capacity relies on DR resources. Power reserve requirements are alleviated by much less operating power reserve resources with DSFR shifting loads and reserving more DR resource capacity.

## Results

### Models and scenarios

**Figure 1:**
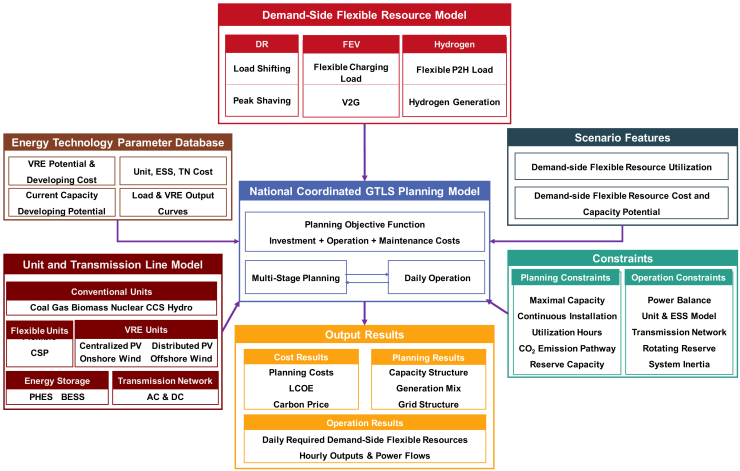
National coordinated generation-transmission-load-storage (GTLS) planning framework Demand-side flexible resource models describe various load regulation capabilities of DR, FEV, and P2H. Energy technology parameter database consists of costs and developing potentials of different elements (VRE and other units, ESS, and transmission network (TN)), as well as load curves. Scenario features include the deployment, cost, and capacity potentials of various DSFR. Unit and transmission line models consist of various conventional units, typical flexible units, VRE units, ESS, and TN. Typical planning and operation constraints are considered in the national coordinated GTLS planning model, which combines multi-stage planning and typical day operations to minimize the overall power system transition cost. Various types of cost results, planning, and operation results, such as planning cost and capacity structure, can be obtained from this model.

DSFR impacts on the power system transition costs and morphology toward carbon neutrality are quantitatively assessed by a national coordinated GTLS planning framework, whose structure is shown in Figure 1. The key part of this framework is the multi-stage coordinated GTLS planning model, which is formulated as a large-scale linear programming optimization problem. We establish detailed component models of generation units, ESS, inter-provincial transmission networks, and various DSFR. Energy technology parameters, load demand and carbon emission budget of each stage are given as boundary conditions.

Our framework focuses particularly on load demands to examine demand-side flexibility. We divide the total load into EV charging, P2H, DR load, and other inflexible load demands. We establish detailed formulations for DR resources, FEV, and flexible P2H resources considering their operation schemes and satisfying their load demand requirements during daily operations. The costs and development potentials of various DSFRs are investigated and further projected. The hourly load curves, EV charging curves, and VRE output curves in different provinces for the period from 2025 to 2060 are obtained as the daily operation boundaries. In order to evaluate the effect of each kind of DSFR on the China’s power system carbon neutrality transition, the scenarios are classified by which type of DSFR is utilized and whether the carbon budget is considered. While the sensitivity analysis considers the cost and capacity potential uncertainty of various DSFR.

The model minimizes total costs, composed of investment, operation, and maintenance costs of different components, including generation units, ESS, inter-provincial transmission networks, and DSFR. The multi-stage planning and typical day operations are combined by coupled constraints such as utilization hour constraints to ensure planning results applicable to system operations. We cluster the 8760-h load curves and VRE output curves into 12 daily curves for each province using the K-medoids method,^61^^,^^62^^,^^63^ which provides boundary conditions for operations of 12 typical days. Furthermore, we consider the costs of some intra-provincial power system development by predicting the capacity of local transformers and networks after optimization, from decision variables of local generation unit capacity and maximum local load in each stage. The detailed parameters, formulated model, and scenario features are shown in the STAR Methods section.

We investigate the current deployment of DSFR and assess their development potentials to incorporate into boundary conditions. These DSFR provide multiple load regulation capabilities for power system operations, such as load shifting, load shaving, power reserve, and operation reserve. The reserve capabilities contribute to power balancing and system stability, especially in a VRE-dominated power system. We account for two types of DR resources: peak-shaving and load-shifting resources. These DR resources, including HVAC systems, industrial loads, 5G stations, and other distributed load resources, are aggregated by load aggregators or virtual power plant (VPP) operators to provide greater power responses. The peak-shaving DR resources cut off peak load when the power supply is inadequate. The load-shifting DR resources reschedule their load demand periods while their daily total load demands remain unchanged. Both of their applicable capacity in each province will reach 5% of the provincial maximum load in each stage.^33^ The flexibly controlled EVs are expected to be capable of adjusting their charging periods (flexible charging load) and providing power outputs (V2G) in the future, which are aggregated into the FEV in each province. Their daily charging load demands, rather than hourly charging load, are satisfied through daily operations. We collect the future P2H load demands and their proportions in the total hydrogen demand, which will reach 1293.6 TWh and account for 70% by 2060.^5^^,^^64^ The flexible P2H resources adjust their hourly load demands (flexible P2H load) while satisfying the annual P2H load demand amount. P2H production is not required to satisfy hourly load curves as hydrogen can be easily stored for a long duration. Moreover, the stored hydrogen can be utilized to provide power outputs (hydrogen generation). supplemental information

We establish an energy technology parameter database with economic parameters, development potentials, hourly load, and VRE output curves. Investment, operation, and yearly maintenance costs are collected to model thirteen types of generation units and two types of ESS with operation attributes such as resource consumption factors. We aggregate the same type of component in each province with aggregated provincial development potentials and capital costs. Inter-provincial transmission lines between the same provinces are merged as one specific transmission line. Load and VRE output curves with 8760 h in each province are clustered into several daily curves for typical day operations. National policies on coal and nuclear units’ development and generator manufacturing capability are involved in this model as constraints. Net present values in 2020 of total investment costs, operation costs, maintenance costs, and intra-provincial transmission network development costs from 2020 to 2060 are summed and further defined as the overall cost.

We design six scenarios to analyze DSFR contributions to the carbon neutrality transition, whose abbreviations and specific settings are shown in Table 1: Business-*as*-usual scenario (BAU, without carbon emission budget and DSFR), Reference scenario (REF, with carbon emission budget and without DSFR), DR scenario (DR, with DR resources), EV scenario (EV, with FEV), P2H scenario (P2H, with flexible P2H resources), and All-demand-flexibility scenario (ADF, with all DSFR). The overall planning cost of the BAU scenario is driven by load growths in the 40 years. The only difference of boundary conditions between the BAU scenario and REF scenario is the carbon emission budget, resulting in additional costs to achieve carbon neutrality in the REF scenario. The overall planning costs of the BAU scenario and REF scenario are compared to quantify how much additional costs will be required for achieving carbon neutrality in China’s power system. DSFR contributions to cost reduction are quantified and analyzed based on additional transition costs. Transition results of the REF scenario, such as capacity structure and generation mix, are set as a reference for comparisons. The DR scenario, the EV scenario, and the P2H scenario are compared with the REF scenario to evaluate the contributions of each type of DSFR. The ADF scenario is set to examine the synergistic effects among different DSFR. supplemental information

**Table 1:** The descriptions and settings of different scenarios

Name	Description	CO_2_ Budget (billion metric tonnes)	DR	EV	P2H
BAU	No carbon emission budget constraint. The transition cost is only caused by load demand growth, taken as the reference for comparison with the REF scenario.	NA	✗	✗	✗
REF	A carbon emission limit pathway from 2020 to 2060 is considered, while no DSFR is introduced. The transition cost differences compared with the BAU scenario show the impact of the carbon neutrality goal. Transition results of the REF scenario are taken as the reference to evaluate the contributions of various DSFR.	105.6^a^	✗	✗	✗
DR	DR resources are introduced to evaluate their influence on the carbon neutrality transition while EV charging load and P2H load demands remain inflexible. Other settings are the same as the REF scenario.	105.6	✓	✗	✗
EV	FEVs are allowed to perform V2G and adjust charging periods to evaluate their influence on the carbon neutrality transition. DR resources and P2H load demands remain inflexible. Other settings are the same as the REF scenario.	105.6	✗	✓	✗
P2H	Flexible P2H resources are introduced to evaluate their influence on the carbon neutrality transition, while both EV charging load and DR resources remain inflexible. Other settings are the same as the REF scenario.	105.6	✗	✗	✓
ADF	DR resources, FEV, and flexible P2H resources are introduced to analyze their synergistic influences on the power system's carbon neutrality transition. Other settings are the same as the REF scenario.	105.6	✓	✓	✓

a The detailed load demand growth in provinces and carbon emission trajectory are derived and further projected based on the Report on China’s “14th Five-Year” Power Development Plan^61^ and other related reports,^62^^,^^65^ shown as Table S1 and Figure S19 in supplemental information.

### Demand-side flexible resources lower carbon neutrality transition costs

**Figure 2:**
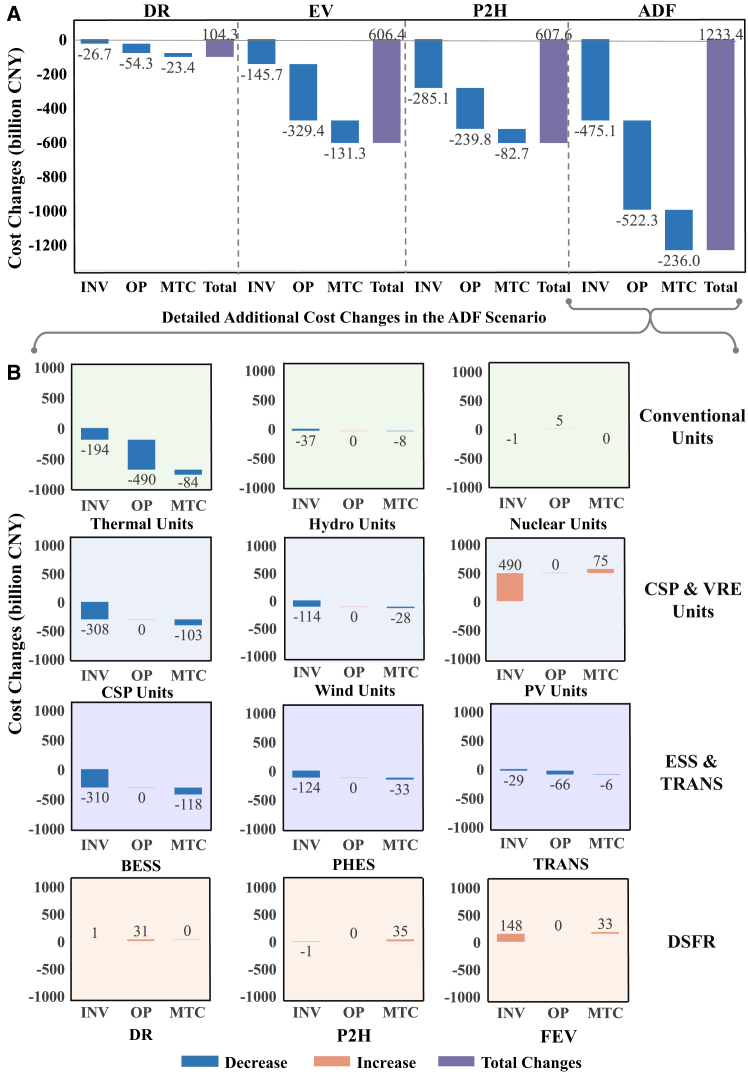
Transition cost changes (A) Transition cost changes between the REF and other scenarios. Three types of transition costs for each scenario are listed: investment cost (INV, capital costs), operation cost (OP, fuel costs and DSFR employment costs), and maintenance cost (MTC, fixed annual operating costs, scheduled maintenance, and overhaul costs). We set the cost of the REF scenario as the reference and compare the same type cost change of between each scenario and REF scenario. Blue bars represent cost decrease, and purple bars represent the total cost changes between each scenario and REF scenario. The figure later in discussion or above each bar is the value of the corresponding cost change. (B) Detailed cost changes of various components between ADF and REF scenario. These cost changes are divided into four groups based on their capabilities. Conventional units include thermal, nuclear, and hydro units. CSP and VRE units include CSP, wind, and PV units. ESS and TRANS include pumped hydro energy storage (PHES), battery energy storage system (BESS), and both inter-provincial and inter-provincial transmission networks (TRANS). DSFR includes demand response resources (DR), flexible P2H resources (P2H), and flexible electric vehicles (FEV). Fewer cost changes are listed on the left in each group.

The overall costs of the BAU, REF, DR, EV, P2H, and ADF scenarios are 44.0 trillion, 50.2 trillion, 50.1 trillion, 49.6 trillion, 49.6 trillion, and 49.0 trillion CNY, respectively. These costs reflect how much the load growth boosts the generation unit development in China’s power system. The overall cost changes between each scenario and the BAU scenario are defined as the additional transition costs, driven by the carbon neutrality goal rather than the load growth. A transition cost of 6.2 trillion CNY is required to achieve carbon neutrality without any DSFR in the REF scenario, considering load growth and carbon emission budget, coming from replacing thermal units with large-scale VRE units and ESS. The full participation of DSFR reduces the cost to 5.0 trillion CNY in the ADF scenario, dropping by 20%, with a DSFR cost of only 0.25 trillion CNY. The reduced transition costs of investment, operation, and maintenance are 0.48 trillion, 0.52 trillion, and 0.24 trillion CNY, respectively (see Figure 2). The costs of thermal units, BESS, and CSP units are greatly reduced when fully deploying DSFR, while more PV units are invested. The investment cost reduction mainly comes from fewer thermal units, CSP units, BESS, and PHES. Besides, the greatly reduced operation cost results from less dispatching thermal units.

DSFR incorporation benefits the diversified deployment of low-carbon resources in a cost-effective manner with synergistic load regulation capabilities. FEV and flexible P2H resources mainly provide flexibility by load shifting during each day, greatly benefiting transition cost reduction with their considerable load demands. DR resources perform load shifting and peak load shaving with fewer transition cost reductions due to limited flexible load demands. DR resources mainly provide power reserve capacity for system security.

Among DSFR, FEV and flexible P2H resources lower the costs of achieving carbon neutrality to a greater extent than DR resources. FEV and flexible P2H resources aid in transition cost reduction by substituting the conventional and flexible units with VRE units and less utilizing ESS to avoid energy losses. The EV scenario transition cost decreases by 9.71%, where the operation cost reduction accounts for 54.3%. The P2H scenario further reduces the required capacity of CSP units, resulting an investment cost reduction of 46.9% of the overall transition cost reduction. In the DR scenario, DR resources make a smaller impact of reducing 1.67% of the transition cost compared with the REF scenario, where the operation cost contributes half of the whole cost reduction with 54.3 billion CNY. These reductions are mostly driven by dispatching more load-shifting DR resources and fewer starting thermal units.

FEV and DR resources both contribute to greater reductions in operation costs, predominantly from thermal units and transmission losses as loads are shifted and managed more locally. Flexible P2H resources are able to reduce investment costs in ESS, thermal and CSP units with around 0.15 trillion CNY. All resources promote the substitution of VRE for conventional generators with fewer investment and operation costs. They further alleviate the dependence on thermal units and ESS, replaced by more PV units of 0.57 trillion CNY.

### Demand-side flexible resources reduce storage and firm capacity requirements

**Figure 3:**
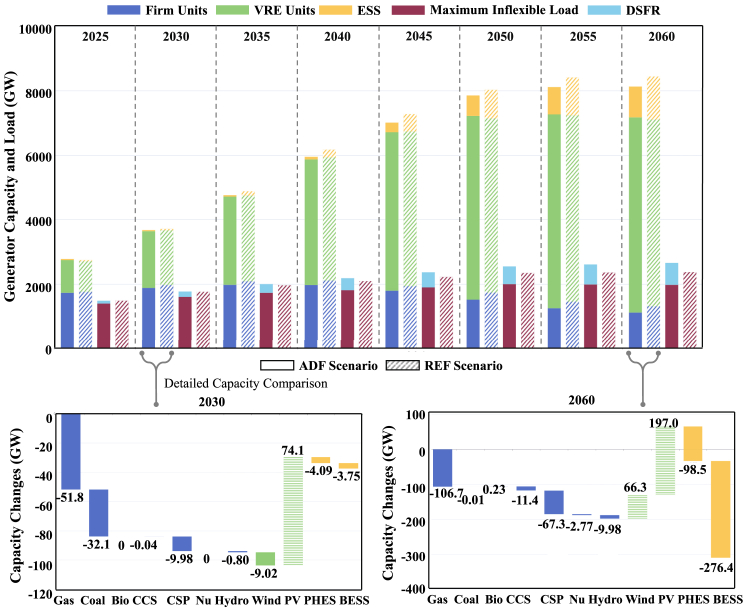
National power supply and power load development pathway comparison between the ADF scenario and the REF scenario We display the power supply capacity and power load from 2025 to 2060 in the ADF scenario (solid color blocks) and the REF scenario (color blocks with diagonals). Power supply bars show the capacity of firm units (blue bars, including coal, gas, biomass, CCS, hydro, nuclear, and CSP units), VRE units (green bars, including wind and PV units), and ESS (yellow bars, including PHES and BESS). Power load bars represent the maximum inflexible load (red bars) and DSFR capacity (light blue bars). The detailed power supply capacity changes of various units and ESS between the ADF scenario and the REF scenario in 2030 and 2060 are displayed in two subfigures. The capacity decrease is represented by solid color blocks, and the capacity increase is represented by color blocks with horizontal lines, where figures above or under bars are values of the corresponding cost changes.

Large-scale VRE installations will be required to replace thermal units to achieve the carbon neutrality transition by 2060, with a large amount of ESS balancing VRE and providing power reserve capacity. VRE capacity will increase from around 3580 GW (BAU) to 5850 GW (REF). More power reserve resources, such as gas and CSP units, will also be required to support safe system operations. The incorporation of DSFR benefits the carbon neutrality transition by reducing ESS and firm unit capacity requirements; see Figure 3A. At the end of the transition, about 1110 GW firm units and 960 GW ESS will be required to achieve carbon neutrality (ADF), decreasing by 200 GW and 375 GW compared with the REF scenario in 2060, respectively.

DSFR can provide a valuable opportunity in the medium term to reduce thermal unit requirements at carbon peaking and accelerate carbon neutrality. Coal, gas, and CCS capacity will greatly decrease at the carbon peaking stage in the ADF scenario, further decreasing 84 GW in total compared with the REF scenario. Their generated energy will be less required as DSFR adjust heavy load periods to accommodate more energy from wind and solar units. The pace of VRE replacing conventional generators is accelerated with the development of DSFR. Compared with the REF scenario, 9.02 GW wind unit capacity will be less required in the ADF scenario in 2030. Whereas, the PV unit capacity will increase 74.1 GW by 8.03% because the replacement of DSFR to power reserve resources leads to more capacity requirements of low-cost PV units. VRE capacity increases from about 5800 GW (REF) to 6060 GW (ADF) in 2060, contributing another 3.3% of total generated energy. Meanwhile, thermal units generate less than 37% of their original generated energy at the carbon neutrality stage, with much fewer carbon emissions due to earlier retirements. Dependence on power reserve resources will be alleviated by DSFR with large flexible load capacity, resulting in much less capacity of ESS and other flexible units such as CSP. Compared with the REF scenario in 2030, only 8.76 GW CSP unit will be required in the ADF scenario, reducing by 53.3%.

### Widely developed demand-side flexible resources assist regional load regulation and VRE accommodation

The rapid growth of DSFR provides considerable flexible capacity for the power system, as shown in Figure 3B. The overall capacity requirements of all DSFR will grow to 680 GW in 2060, composed of 302 GW FEV, 284 GW flexible P2H resources, 51 GW load-shifting and 43 GW peak-shaving DR resources. DSFR provides the second largest flexible capacity among all controllable resources at the carbon neutrality stage, only 63 GW less than BESS. The flexible load proportion (DSFR controlled load over the peak load) increases to 25.6% by 2060, greatly alleviating power balancing burdens for the decarbonized power system. Load energy regulations mainly rely on adjusting load curves of FEV and flexible P2H resources, whose shifted load demands account for more than 99.8% of the whole flexibly controlled load demands. Although with a small share of shaved and shifted load demands, DR resources provide considerable power reserve capacity as critical reserve resources.

**Figure 4:**
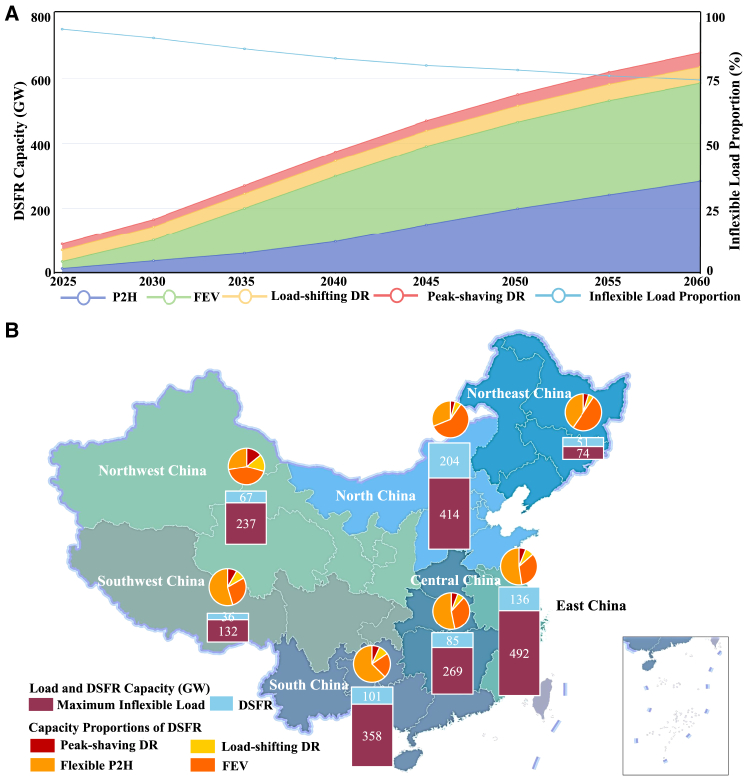
DSFR development pathway and regional distribution in China (A) DSFR development pathway and maximum inflexible load proportions from 2025 to 2060 in the ADF scenario. The capacity of various DSFR from 2025 to 2060 are displayed, including flexible P2H resources (P2H), flexible EV (FEV), load-shifting DR resources (Load-shifting DR), and peak-shaving DR resources (Peak-shaving DR). The maximum inflexible load proportion is the proportion of the maximum inflexible load in the overall load. These inflexible load proportions from 2025 to 2060 in the ADF scenario are represented by the light blue broken line. (B) Regional load control capability and DSFR capacity structure in 2060. We divide Chinese mainland provinces into seven regions: Northeast China, North China, East China, Central China, South China, Southwest China, and Northwest China, with different background colors. The stacked bars represent the maximum inflexible load (red bars) and DSFR capacity (light blue bars). Pie chart sectors represent capacity proportions of various types of DSFR capacity, including peak-shaving DR, load-shifting DR, flexible P2H, and FEV.

Widely deployed DSFR regulates regional load based on regional potentials and power system requirements. Their regional capacity structure and load control capability in 2060 are shown in Figure 4A. DSFR occupies more than 28% of the total load capacity in load-center regions with large potentials, such as North China and South China, mostly composed of FEV and flexible P2H resources. Due to the lack of large-scale VRE in these regions, DSFR flexibly shifts load demands to consume more PV and wind energy from western regions and avoid operating local thermal units frequently. While in western regions with much more VRE capacity but fewer load demands, DSFR provides essential balancing and reserve capacity to alleviate VRE power variations for export. Though with lower flexible load proportions, western regions require larger shares of load reserve resources. In Northwest China, DR resources occupy 29.5% of the total DSFR capacity to provide instant power response and controllable reserve capacity.

### Demand-side flexible resources provide low-cost reserve capacity options for power system operations

**Figure 5:**
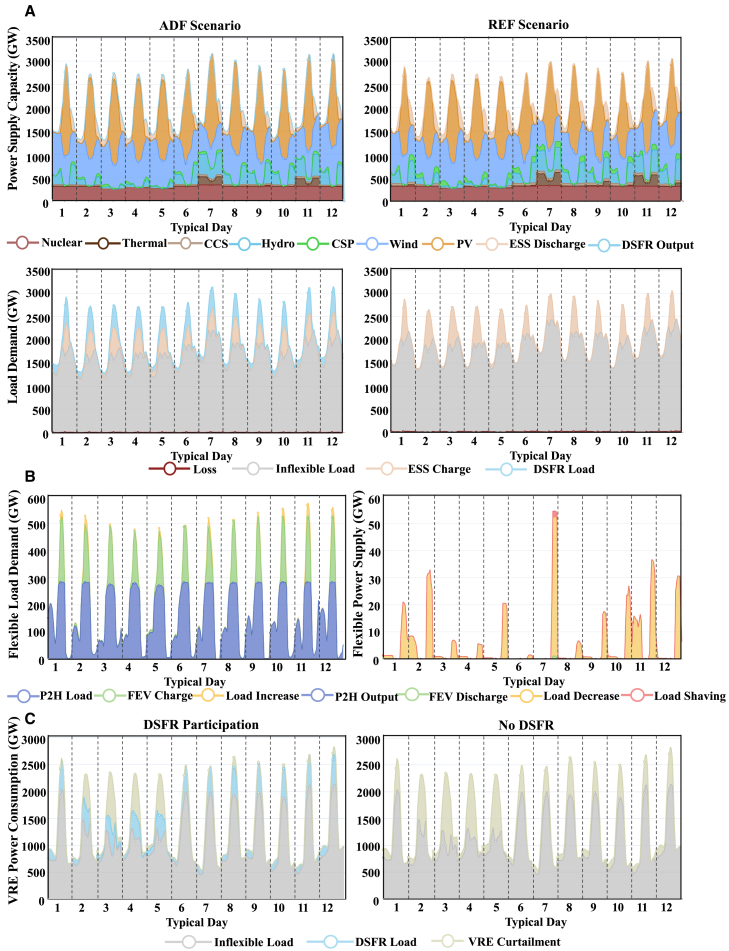
Typical day operation comparison between the ADF scenario and REF scenario in 2060 (A) Hourly power supply and load demands in the ADF scenario and REF scenario. The top two subfigures display stacked power outputs of various components in twelve typical days, including nuclear units, thermal units (except for CCS units), CCS units, hydro units, CSP units, wind units, and PV units. ESS discharging power output (ESS Discharge) and DSFR power output (DSFR Output) are also included. The bottom subfigures display various power demands, including inter-provincial transmission network loss (Loss), inflexible load, ESS charging load demand (ESS Charge), and DSFR load demand (DSFR Load). The power supply capacity and load demands are balanced at each time point during all typical days. (B) Hourly DSFR load demand and power supply in the ADF scenario. The left subfigure displays the hourly load demands of DSFR, including loads of flexible P2H resources (P2H Load), charging loads of FEV (FEV Charge), and load increases of load-shifting DR resources (Load Increase). The right subfigure displays the power supplies of different DSFR, including the flexible P2H resource power outputs (P2H Output), FEV discharging power (FEV Discharge), load decreases of load-shifting DR resources (Load Decrease), and shaved load of peak-shaving DR resources (Load Shaving). It should be noticed that the two subfigures have different y axis scales for the convenience of showing power outputs and load demands of various DSFR. (C) Hourly VRE power output accomodation comparison in the ADF scenario. The left subfigure displays hourly VRE power output accomodation with DSFR, divided into curtailed VRE power (VRE Curtailment), DSFR load, and other inflexible load supplied by VRE (inflexible Load). The right subfigure displays hourly VRE power output accommodation (if without DSFR participation) in the ADF scenario, divided into curtailed VRE power (VRE Curtailment) and other inflexible load consumed by VRE (inflexible Load).

DSFR provides low cost reserve capacity options to less expensive power reserve resources during system operations. Power system operation requires controllable resources, such as CSP, hydro, online operated thermal units, and ESS, to provide instant power response, namely, rotating power reserve. However, these power reserve resources are usually come with high costs or are limited by emission budget constraints during operations. DSFR replaces traditional power reserve resources and provides low-carbon controllable reserve capacity. Detailed hourly power supply and load are shown in Figure 5A.

FEV and flexible P2H resources reduce the rotating power reserve capacity requirements by lowering load demands when power reserve resources are inadequate, such as in the evening and early morning (see Figure 5B). In these periods, both controllable units and ESS provide higher power outputs due to relatively low PV power outputs, resulting in much less applicable rotating power reserve capacity. With FEV and flexible P2H resources, ESS discharging power can be decreased by 117 GW. Their flexible load demands will be rescheduled during daytime to consume more VRE energy and alleviate VRE curtailments with less operating conventional units, as shown in Figure 5C. Additional 375 TWh VRE energy will be consumed more in 2060 compared with the REF scenario, and the curtailed VRE energy decreases by around 75%.

Compared with FEV and flexible P2H resources, DR resources provide essential power reserve capacity and fast power responses. Peak-shaving DR resources only shave their load during critical time periods with insufficient power generation. While load-shifting DR resources averagely operate 94.5% of their capacity for load shifting with other remained capacity for reserve.

### Sensitivity analysis

We focus on both cost and capacity potential uncertainty of DSFR and perform sensitivity analyses based on the ADF scenario to explore their influences on transition cost, LCOE, and capacity structure in 2060. These uncertainties come from the technological improvement, load growth, as well as from electricity market reforms. Starting from the cost settings of each DSFR in the ADF scenario, we vary them within the range of [-40%,40%] with a step size of 10%. For DR resources, we set two scenarios with lower and higher capacity potentials represented by Low DR and High DR scenarios. DR resource potentials are set as 2% (Low DR) and 10% (High DR) of the maximum load. For both EV and P2H resources, we set six scenarios to represent their load demand uncertainties driven by policies and technology improvements, represented by EL (Extra Low), Low, SL (Slight Low), SH (Slight High), High, and EH (Extra High). Their capacity potentials of these scenarios for EV and P2H are set by incorporating their projected load demands from 2025 to 2060 supplemental information.^66^^,^^67^^,^^68^^,^^69^^,^^70^ Other load demands are adjusted to remain the same overall load demands as the ADF scenario.

**Figure 6:**
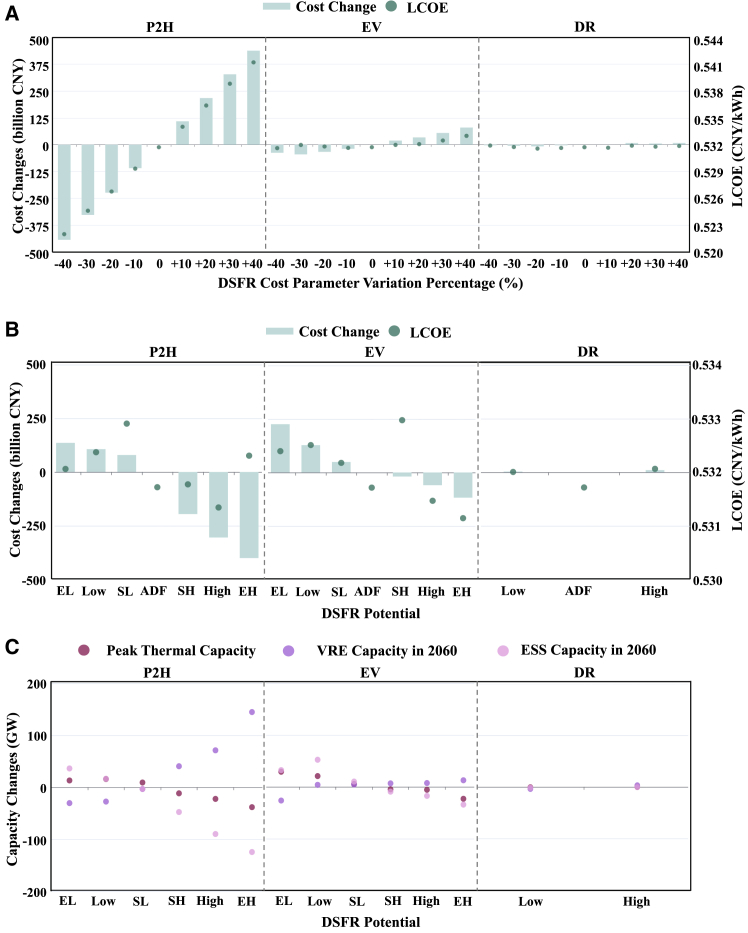
DSFR cost and potential sensitivity analysis comparison (A) Transition cost changes and LCOE with DSFR cost variations. We vary costs of flexible P2H resources, FEV, and DR based on the ADF scenario to design another eight scenarios for each type of DSFR. The horizontal axis represents the relative variation proportion for each cost. (B) Transition cost changes and LCOE with various DSFR capacity potentials. We change the capacity potentials and load demands of P2H and FEV, and the maximum capacity of DR based on the ADF scenario. Transition cost changes set the transition cost of the ADF scenario as a reference. For P2H and FEV, we set extra low (EL), low, slight low (SL), slight high (SH), high, and extra high (EH) scenarios. For DR, we set low and high scenarios. (C) Capacity changes with various DSFR capacity potentials. We display the peak thermal capacity changes in 2035 (including coal, gas, biomass, and CCS capacity), VRE (including PV and wind units), and ESS capacity changes in 2060 with various DSFR capacity potentials. For P2H and FEV, we set extra low (EL), low, slight low (SL), slight high (SH), high, and extra high (EH) scenarios. For DR, we set low and high scenarios. Capacity results of the ADF scenario are set as a reference for comparison.

The cost uncertainty of flexible P2H resources has significant influences on transition costs, while those of FEV and DR resources show much weaker impacts, as shown in Figure 6A. Around 0.43 trillion CNY will be reduced with a 40% decrease of P2H costs, further lowering the carbon neutrality transition cost from 20% (ADF) to 27%. The maximum transition cost changes of FEV and DR resources only account for 1.3% and 0.13% of the original carbon neutrality transition cost, respectively. The larger capacity potential of P2H resources leads to more transition cost reduction of around 6.4%, as shown in Figure 6B. Capacity potential changes of FEV have a slighter influence on transition costs, especially with larger FEV potentials such as the EH EV scenario. Whereas those of DR resources only have minor impacts on transition cost and LCOE.

Larger capacity potentials of FEV and P2H resources accelerate thermal units retirement and VRE replacement, as well as alleviating dependence on ESS. In the EH P2H scenario, the peak thermal capacity (in 2035) drops around 38.5 GW, and the VRE capacity (in 2060) will increase by 144.5 GW. While larger EV capacity has fewer contributions on VRE installations, whose capacity only increases by 12.6 GW in EH EV scenario. The substitution effects between DSFR and ESS will lead to much less ESS capacity in 2060 with larger potentials of FEV and P2H resources. Over 124 GW ESS will be less required in the EH P2H scenario because of the higher penetration of flexible P2H resources. The potential capacity changes of DR resources have small influences on the capacity structure, further highlighting their roles of critical power reserve resources.

## Discussion

Transitioning to carbon neutrality by 2060 would increase the cost of China’s power system by a total of 6.2 trillion CNY. The participation of DSFR with untapped potentials can help reduce these costs but is relatively underexplored in analyses on the carbon neutrality transition. DSFR greatly benefits the power system transition cost reduction by around 20%, providing flexibility for load-shifting, peak-shaving, and system support. Interactions between generation units and load demands contribute to lowering conventional unit installations and power system operations, especially in the carbon neutrality stage. These flexible resources, along with better technical-economic performance, will play a major role in China’s power system transition and influence the development pathways of generation units and ESS.

DSFR can substitute for ESS and other flexible resources, such as gas and CSP units. Compared with other flexible resources, DSFR mainly provided flexibility with their load balancing and system support capabilities. When considering DSFR, the required ESS capacity in 2060 falls by around 28%. The required flexible resources will be more accurately estimated after incorporating the potentials and operation characteristics of DSFR. The greatest benefits come from FEV and flexible P2H resources due to their larger load adjustment potentials among all DSFR. Some regions with lower projected EV and P2H load demands have an important reliance on DR resources.

DSFR contributes most in terms of load-shifting as compared to peak load shaving, highlighting the need for managing energy demand and VRE supply over the day. DSFR increases VRE power generation by 3.4% and reduces CSP and gas generation by 52% and 42%, respectively, in 2060. System reliability is supported by DSFR through controllable reserve capacities, reducing requirements for large backup generator supplies such as thermal units. In the medium-term period (out to 2030), DSFR benefits reduce thermal generation requirements. DSFR thereby reduces long-term lock-in of more than 30 GW coal units that will need to retire by 2060 and reduces at most 195 GW gas unit requirements after carbon peaking.

The uncertainty of DFSR costs has a large influence on carbon neutrality transition costs, most notably flexible P2H resources. The P2H demands in the future will be strongly driven by overall hydrogen demand. It is also affected by how fast P2H substitutes conventional hydrogen production processes such as steam reforming or methane reforming. The growing P2H demands (if flexible) help reduce VRE generated energy curtailment and depend on the reduced expenses and advanced technologies. Their technology development highly influences the deployment of DSFR by changing the projected costs, which may boost or set back the promotion in the future. More comprehensive analyses on load demand potential and cost changes driven by policies and technologies should be focused on and further examined. New-type load resources with considerable load potentials, such as intelligent computing centers and 5G/6G base stations, may further boost capacity structure decarbonization when fully participating in power system operations. Their response scheme still remains unclear currently, its influences should be further analyzed based on specific schemes.

DSFR development is a long-term and comprehensive challenge. It is not fully determined by the technical economy, which also involves policies and market development. The business model of DSFR has not been fully established in China. The obtained development pathways of DSFR assist governments and industries in putting forward power system decarbonization transition from the demand side. Different economic and social factors, such as consumer willingness and market incentives, also influence the development of DSFR. China’s ongoing electricity market reform will greatly influence the cost of employing DSFR and their flexibility contribution, whose market schemes also influence the participation willingness of DSFR. The market schemes should be capable of pricing DSFR load regulation capabilities to avoid decreasing potentials from mismatch. While DSFR has shown significant contributions to China’s power system carbon neutrality transition, the impacts of DSFR can be quantified and further analyzed based on other continent-scale power system conditions using our method.

### Limitations of the study

The operational characteristics of each type of DSFR are greatly influenced by specific customer behaviors and response schemes in different DSFR programs. More detailed models should be considered when analyzing their practical load regulation contributions to power system operations. Their economic feasibility is also determined by electricity market schemes and policy settings, reflecting DSFR’s response willingness. Although the load regulation capabilities can be quantified by detailed DSFR models and load potential assessment, their economic profits determine whether they will provide load regulations if necessary. Policies and market uncertainties should be further addressed in future research.

## Resource availability

### Lead contact

Requests for further information and resources should be directed to and will be fulfilled by the lead contact, Ning Zhang (ningzhang@tsinghua.edu.cn).

### Materials availability

This study did not generate new materials.

### Data and code availability

• Data: All result datasets in this study can be found at Mendely (https://data.mendeley.com/datasets/y5zczp74rb/2).
• Code: This article does not report the original code.
• All other items: Any additional information required to reanalyze the data reported in this article is available from the lead contact upon request.

## Acknowledgments

This work was supported by 10.13039/501100012166National Key R&D Program of China (2022YFB2403300), the State Key Program of 10.13039/100014717National Natural Science Foundation of China (52130702), the 10.13039/100014717National Natural Science Foundation of China (No. 52177093), Scientific & technical project of State Grid Shanghai Electric Power Company (SGSHDK00DWJS2310470), and Organized Research Support Program, Department of Electrical Engineering, Tsinghua University.

## Author contributions

H.W., N.Z., Z.Z., and C.K. conceived and designed the research. H.W., N.Z., E.D., and Z.Z. formulated the theoretical model. H.W. and Z.Z. conducted the code implements and carried out the simulations. H.W., N.Z., and M.R.D conducted the data analysis and led the writing of the article. H.W., N.Z., H.J., Z.Z., and J.X. contributed to the data collection. N.Z, E.D, H.J, M.R.D, P.W, and W.L contributed to the figure drawing. H.W, N.Z., M.R.D, and C.K. conducted the policy analysis. All authors contributed to the discussions on the framework and the editing of this article.

## Declaration of interests

The authors declare no competing interests.

## STAR★Methods

### Key resources table

**Table:** 

REAGENT or RESOURCE	SOURCE	IDENTIFIER
**Deposited data**		

Cost, capacity, and hourly power output results	Mendeley Data	https://data.mendeley.com/datasets/y5zczp74rb/2

**Software and algorithms**		

MATLAB R2020b	Matlab Software Foundation	https://www.mathworks.com/
Gurobi 9.0.1	Gurobi Optimization	https://www.gurobi.com/
Python 3.7	Python Software Foundation	https://www.python.org/

### Method details

#### National coordinated GTLS planning model

We formulate the national coordinated GTLS planning model with DSFR to explore the Chinese power system transition process from 2025 to 2060. The model accounts for the provincial development of various power system components and the development of inter-provincial transmission networks. Thirteen types of generation units are categorized into conventional units (coal, gas, biomass, nuclear, CCS, and hydro units), flexible units (CSP units), and VRE units (PV and wind units). Their operation attributes consist of carbon emission factors, resource consumption factors, and inherent characteristics such as inertia and ramp rates. ESS consists of pumped hydro energy storage (PHES) and battery energy storage systems (BESS), whose operation attributes include charging/discharging efficiency, inertia and reserved capacity ratios. Both alternating current (AC) and direct current (DC) transmission networks are modeled utilizing the transportation model, whose attributes include their capacity and loss rates. In the inter-provincial transmission networks, we model the extra-high-voltage alternating current (EHVAC), extra-high-voltage direct current (EHVDC), ultra-high-voltage alternating current (UHVAC), and ultra-high-voltage direct current (UHVDC) transmission networks. We aggregate the same type of units or ESS in each province with aggregated capacity, while different inter-provincial transmission lines between the same provinces are merged as one equivalent transmission line. For each of these components, we establish its cost function considering investment, operation, and maintenance costs with detailed costs. Their investment costs, operation costs, and maintenance costs constitute the optimization objective function, while the optimized results are utilized to further analyze the additional costs of intra-provincial transmission networks and distribution networks.

Multi-stage planning and hourly typical day operation dispatch in each stage are integrated into this model. Considering the long-term planning period, we divide forty years into eight stages with eight typical years to perform multi-stage planning, where planning results of the typical year represent the average results of the five years in each stage. We set twelve typical days for each stage to assess the hourly power dispatch results in the operation stage. We utilize the K-medoids clustering method to obtain hourly load charging curves and VRE output curves of typical days from the 8760-hour curves. Planning constraints consider the carbon emission pathway, maximum capacity potential, natural resources adequacy, planning reserve requirement, and installation coupling at different stages. Planning decision variables limit the power system operation through operation constraints. Operation constraints include the rotating reserve capacity constraints, power balancing constraints, power system inertia constraints, and various component models. Operation decision variables of typical days restrict the planning decision variable optimization through utilization hour constraints as well. More detailed planning constraints and operation constraints are displayed in the supplemental information (Note S1).

#### DSFR modeling

The DR resources, FEV, and flexible P2H resources are modeled based on their characteristics and economic parameters. We model two types of DR resources based on different response schemes: peak-shaving DR resources and load-shifting DR resources. The peak-shaving DR resources are capable of cutting off their load during daily operations. We model their investment costs considering the value of their reserved power capacity for peak-shaving and model their operation costs to pay for the shaved load demands. Load-shifting DR resources are able to adjust their load demand periods, which shifts part of their load demands to other periods of the day. The maximum shifted capacity determines the investment costs and the shifted load demands determine the operation costs. The maximum capacity of either peak-shaving DR resources or load-shifting DR resources should not exceed 5% of the maximum load in each province.

We consider the future development of the power system, hydrogen system and transportation system to determine the boundary conditions for FEV and flexible P2H resources. Future load demands are divided into P2H load demands, EV charging load demands, and other load demands. We investigated the existing EV populations and projected their amounts in the future, which is shown in the supplemental information (Table S2). The EV populations determine their load demands, maximum charging capacity, and their energy storage capacity. We generate the 8760-hour EV charging load curves in all provinces according to their EV populations and actual charging curves obtained from field investigation. We model FEV with two capabilities: charging load curve adjustment and V2G discharging. The daily energy demands of FEV remain unchanged, while their charging and discharging behaviors are flexibly controlled by smart charging piles. The charging piles should be retrofitted to be compatible with V2G technology, which is considered in the investment costs of introducing FEV. We consider the flexibly charging behaviors and discharged energy of FEV in their maintenance costs and operation costs, respectively. In addition, inflexibly controlled EV groups still follow their original charging curves.

We collect the current hydrogen demand in each province and investigate the future hydrogen demand growths. As hydrogen demand growth requires more supply from P2H resources, we estimate the P2H supply proportions to determine the P2H load demands. We model the flexible P2H resources with two capabilities: P2H load curve adjustment and power generation using hydrogen. As it is feasible to store and transmit hydrogen on a large scale, the flexible P2H resources only satisfy the yearly hydrogen demands rather than specific load curves. The operation costs take their generated energy from hydrogen into consideration.

#### Cost and operational parameters

We establish an energy technology parameter database with economic parameters, development potentials, hourly load and VRE output curves. The economic parameters of generation units, ESS, and transmission networks are collected from the annual technology baseline (ATB) of NREL and reports from the China Electricity Council, which are classified into investment costs, operation costs, and yearly maintenance costs.^71^^,^^72^ We evaluate the provincial VRE potential and development costs based on the GIS and weather data in GREAN database. The development of coal and nuclear units is restricted by national policies and only a few provinces are allowed to invest in these units with capacity limitations in the future.

We collect and estimate costs and operational parameters of all components. Costs include the capital investment cost, operation cost, and maintenance cost, collected from policies and reports (see Note S3 in supplemental information). The costs of FEV are estimated and collected from reports of pilot projects. The investment cost and maintenance costs of flexible P2H resources are collected from reports.^73^ Operational parameters include the maximum developable capacity, utilization hour bounds, and other critical operation parameters, collected and estimated from reports and GREAN database. Load demands and carbon emission budgets are critical boundary conditions in this model. We divide the load into the EV charging load, P2H load and other load. For each type of load, we investigate its provincial load demands in each stage and typical load curves.^5^^,^^61^^,^^62^^,^^63^^,^^65^^,^^74^ A specific carbon emission pathway for each stage is given based on “14th Five Year Plan” and China’s Long-term Low-carbon Development Strategy and Pathway Report.^33^^,^^62^

The transition cost in the BAU scenario is only caused by load demand growth without any carbon emission budget. All of the other scenarios have the carbon emission budget constraint, whose transition costs are influenced by both load growth and carbon emission budget. Hence, the transition cost differences between other scenarios and the BAU scenarios are defined as the additional transition costs for these scenarios. Lower transition costs are obtained in the DR, EV, P2H, and ADF scenarios as DSFR provide flexibility to assist power system transition. Hence, DSFR contributions are evaluated based on how much they lower the additional transition cost caused by the carbon emission budget.

### Quantification and statistical analysis

There are no quantification or statistical analyses to include in this study.

## Supplemental information

Document S1. Fgures S1–S20 and Tables S1–S4
