# Supplementary material for: Emerging demand-side flexible resources accelerate China’s power system transition toward carbon neutrality
Source: iScience. 2025 Apr 8;28(5):112372. doi: 10.1016/j.isci.2025.112372 (PMC12063150; doi:10.1016/j.isci.2025.112372)
Supplement: Document S1. Fgures S1–S20 and Tables S1–S4 [file mmc1.pdf]

iScience, Volume *28*

Supplemental information

Emerging demand-side flexible resources accelerate China's power system transition toward carbon neutrality

Hongyi Wei, Ning Zhang, Ershun Du, Haiyang Jiang, Zhenyu Zhuo, Michael R. Davidson, Weiran Li, Peng Wang, Jinyu Xiao, and Chongqing Kang

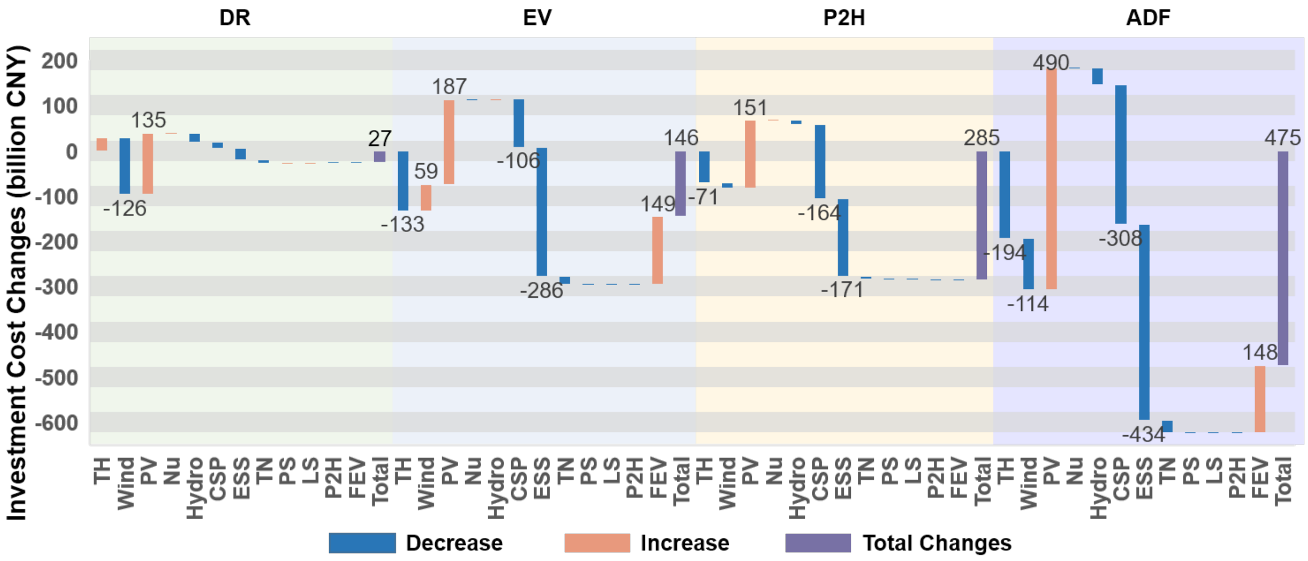

Supplemental Figure 1. Investment cost changes of different components between different scenarios. We set the costs of the REF scenario as the reference. The investment costs changes of different components are listed: thermal units (TH, including coal, gas, biomass and CCS units), wind units, photovoltaic units (PV), nuclear units (Nu), hydro units (Hydro), concentrated solar power units (CSP), energy storage systems (ESS), transmission networks (TN), peak-shaving DR resources (PS), load-shifting DR resources (LS), flexible power-to-hydrogen resources (P2H), and flexible electric vehicles (FEV).

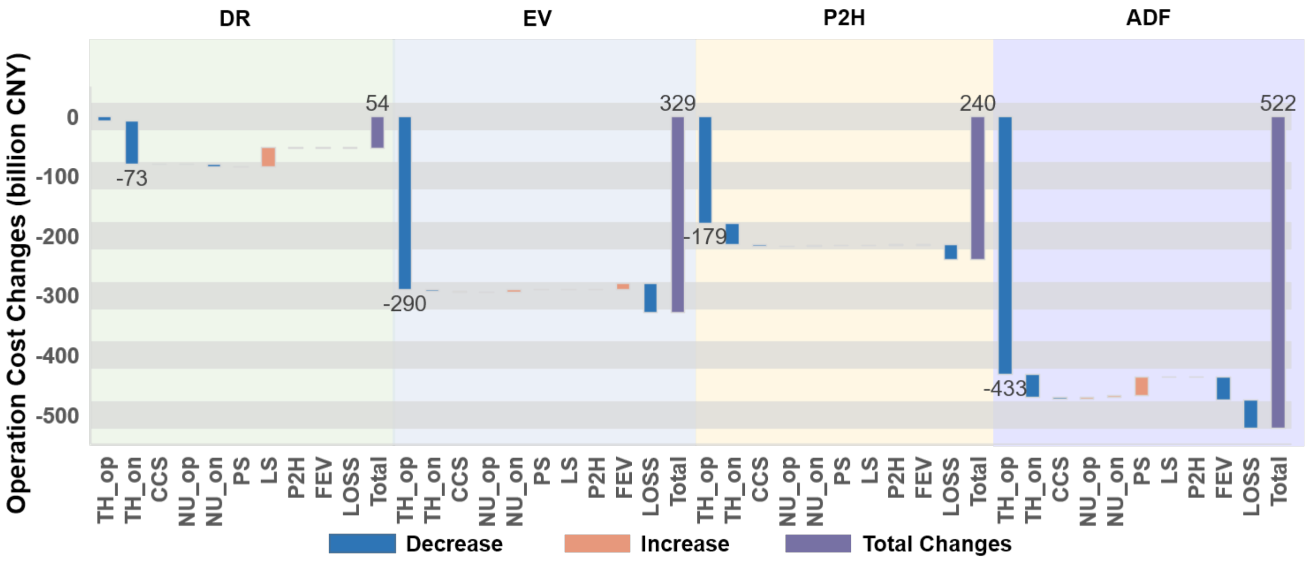

Supplemental Figure 2. Operation cost changes of different components between different scenarios. We set the costs of the REF scenario as the reference. The operation costs changes of different components are listed: thermal units operation cost (TH_op), thermal units start-up cost (TH_on), carbon capture and storage cost (CCS), nuclear units operation cost (NU_op), nuclear units start-up cost (NU_on), peak-shaving cost (PS), load-shifting cost (LS), flexible power-to-hydrogen generation cost (P2H), and flexible electric vehicles discharging cost (FEV).

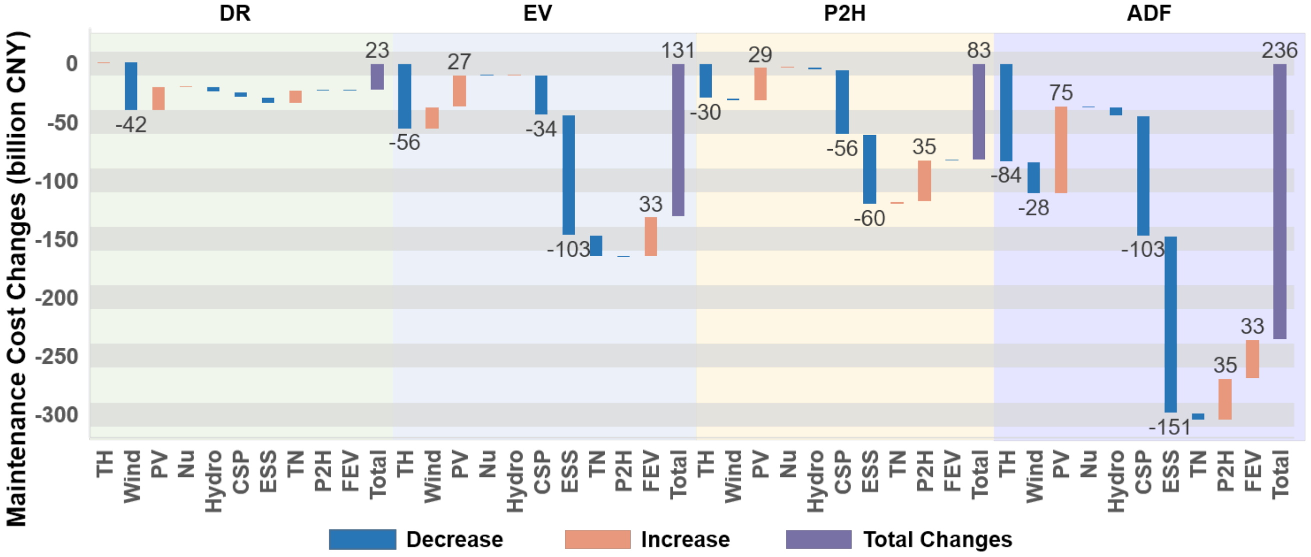

Supplemental Figure 3. maintenance cost changes of different components.

We set the costs of the REF scenario as the reference. The maintenance costs changes of different components are listed: thermal units (TH), wind units, photovoltaic units (PV), nuclear units (Nu), hydro units, concentrated solar power units (CSP), energy storage systems (ESS), transmission networks (TN), peak-shaving DR resources (PS), load-shifting DR resources (LS), flexible power-to-hydrogen resources (P2H), and flexible electric vehicles (FEV).

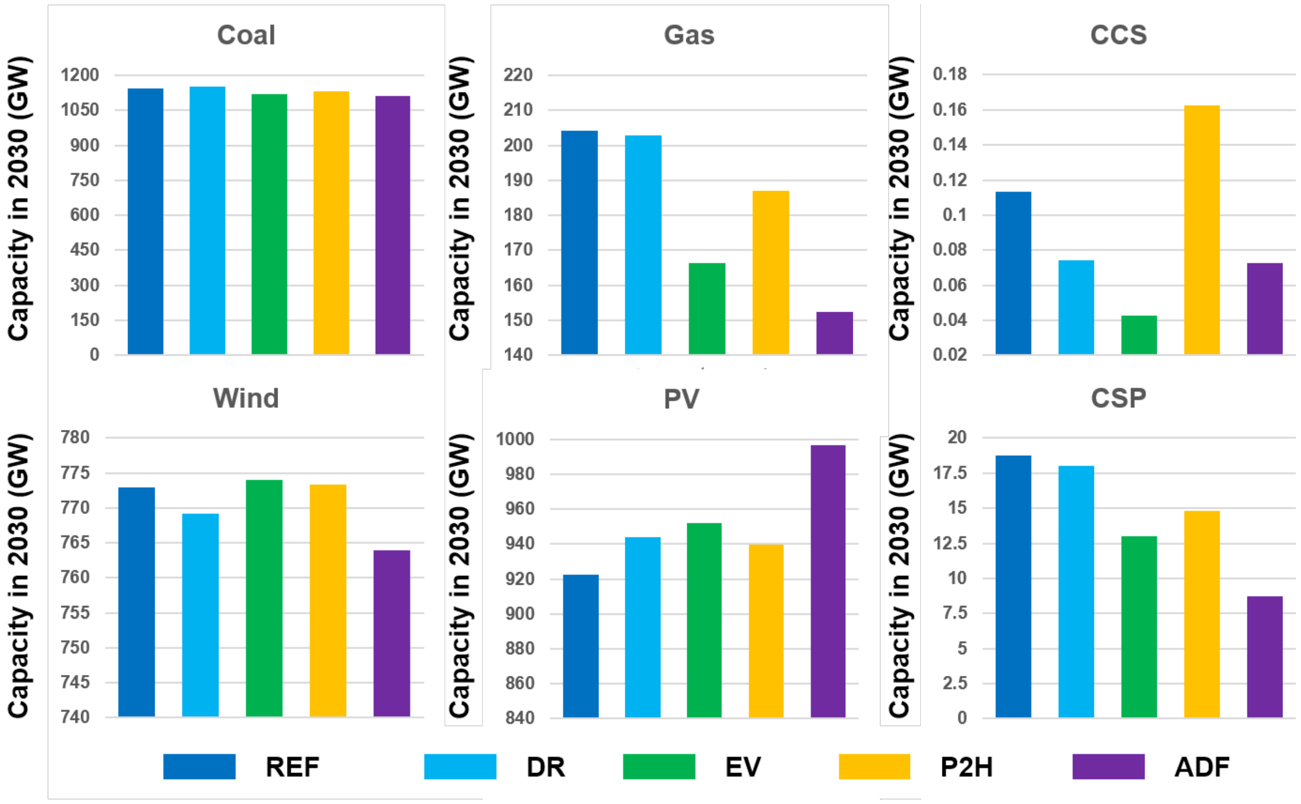

Supplemental Figure 4. Generation unit capacity in 2030 of different scenarios.

We display the capacity of coal units (Coal), gas units (Gas), CCS units (CCS, including coal-ccs units, gas-ccs units, and biomass-ccs units), wind units (Wind), PV units (PV), and CSP units (CSP). The capacity of different scenarios are displayed with different colors. The capacity of nuclear units, hydro units, and biomass units have small differences between scenarios and are not displayed in this figure.

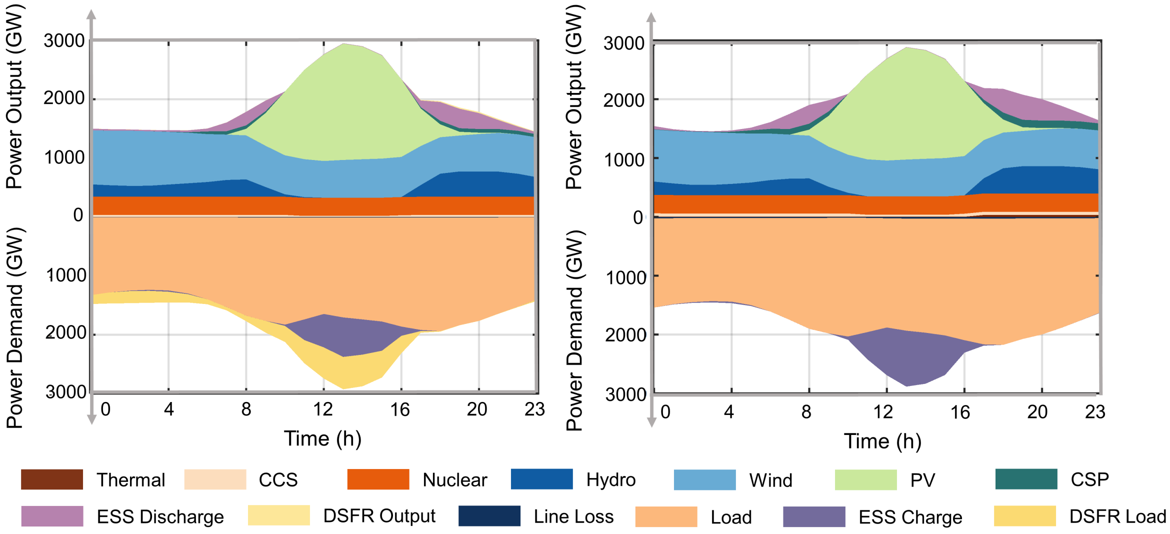

((a)) Hourly operation results of the typical day 1 in the ADF scenario and REF scenario

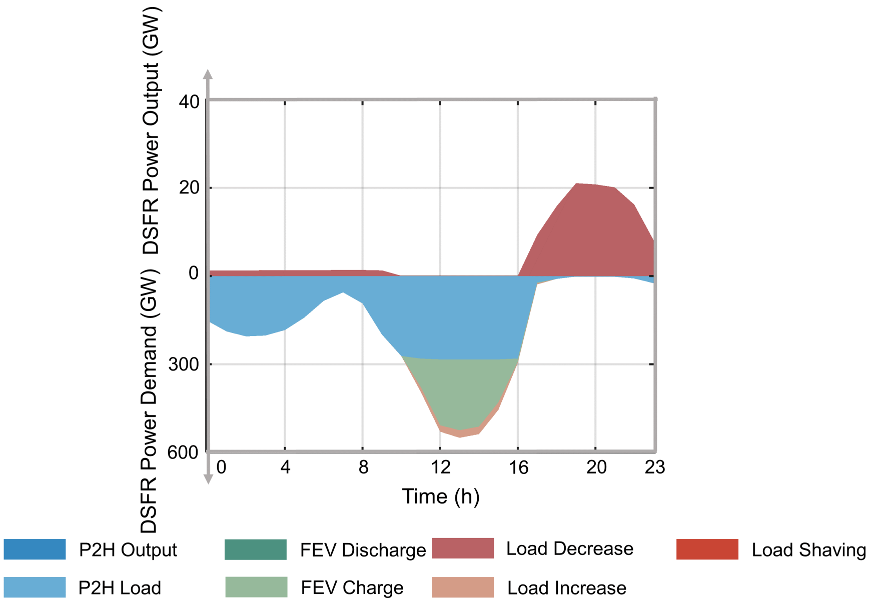

((b)) Hourly operation results of DSFR of the typical day 1 in the ADF scenario

Supplemental Figure 5. Hourly operation comparison in the typical day 1.

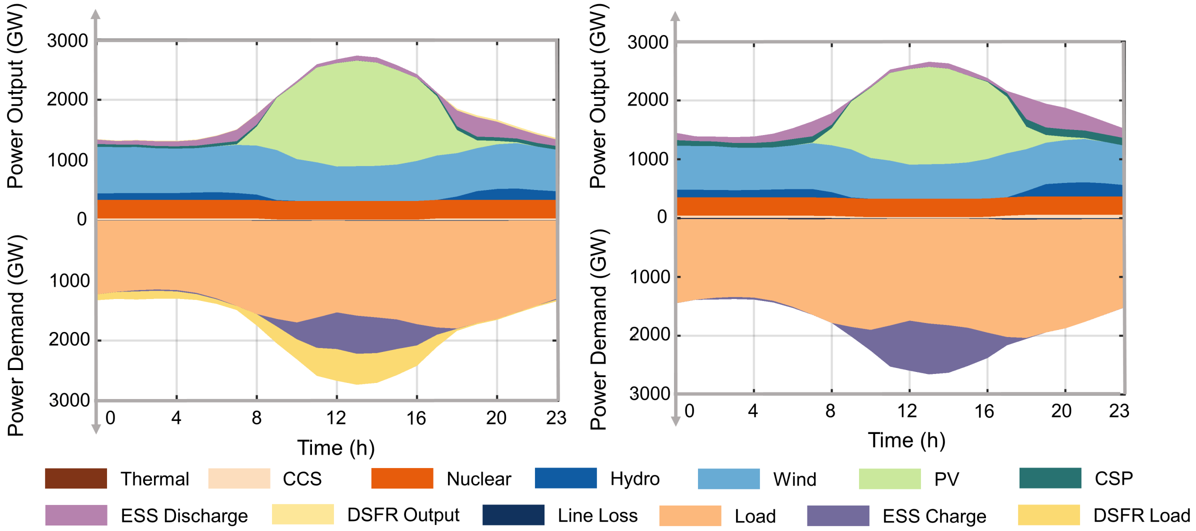

((a)) Hourly operation results of the typical day 2 in the ADF scenario and REF scenario

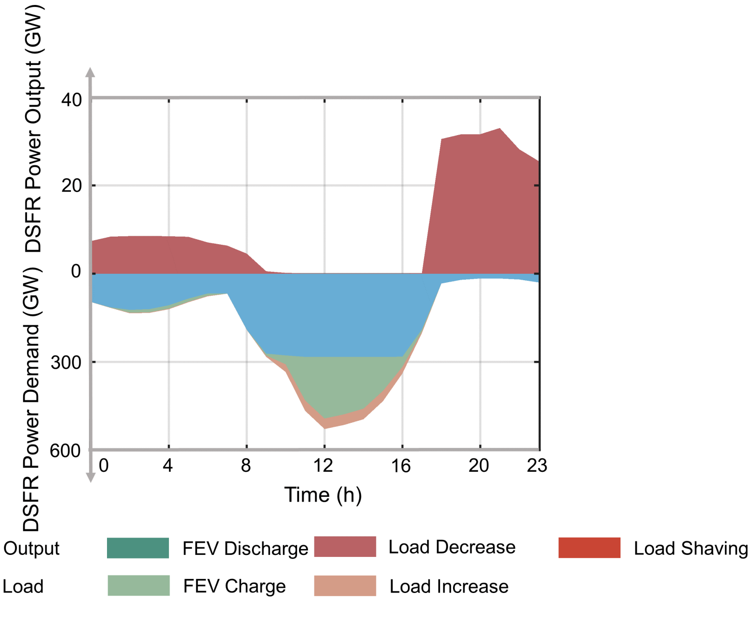

((b)) Hourly operation results of DSFR of the typical day 2 in the ADF scenario

Supplemental Figure 6. Hourly operation comparison in the typical day 2.

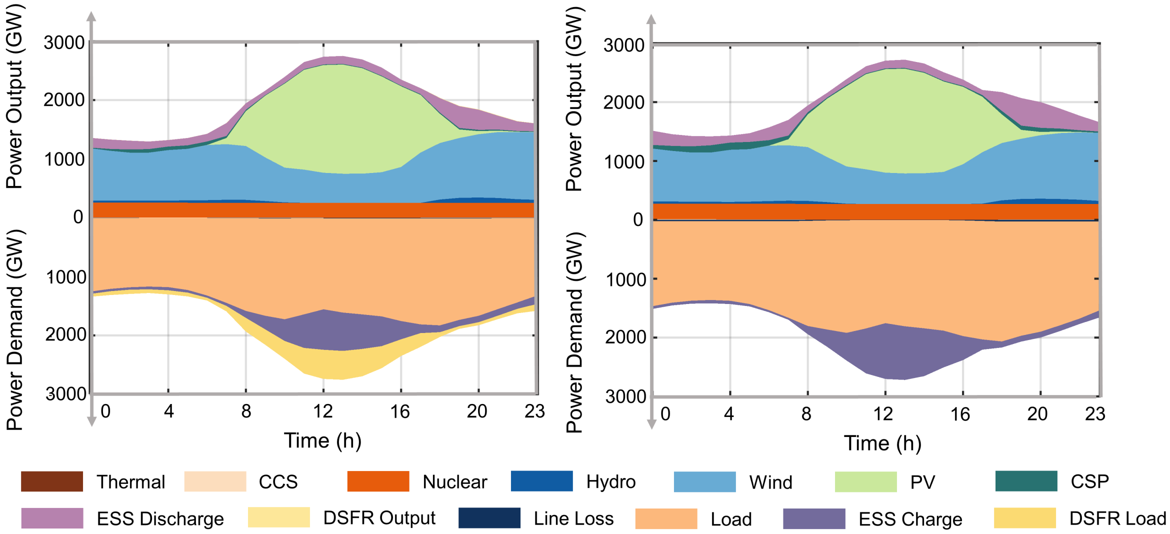

((a)) Hourly operation results of the typical day 3 in the ADF scenario and REF scenario

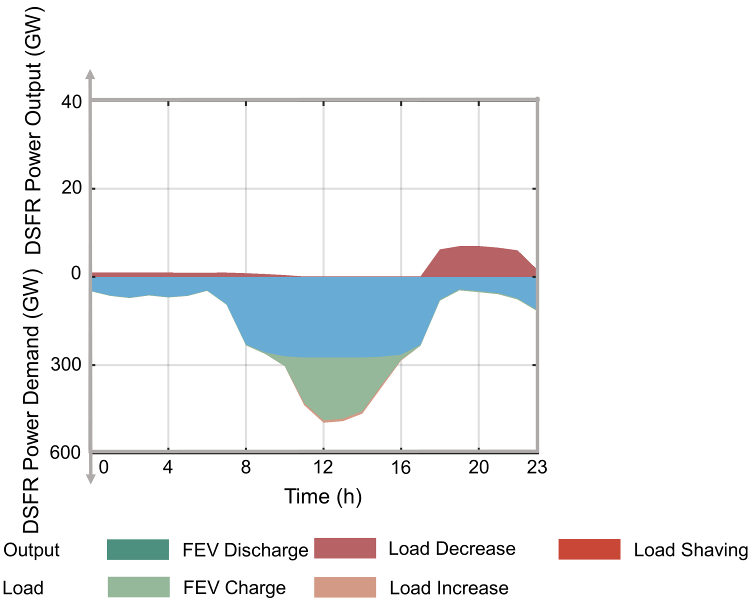

((b)) Hourly operation results of DSFR of the typical day 3 in the ADF scenario

Supplemental Figure 7. Hourly operation comparison in the typical day 3.

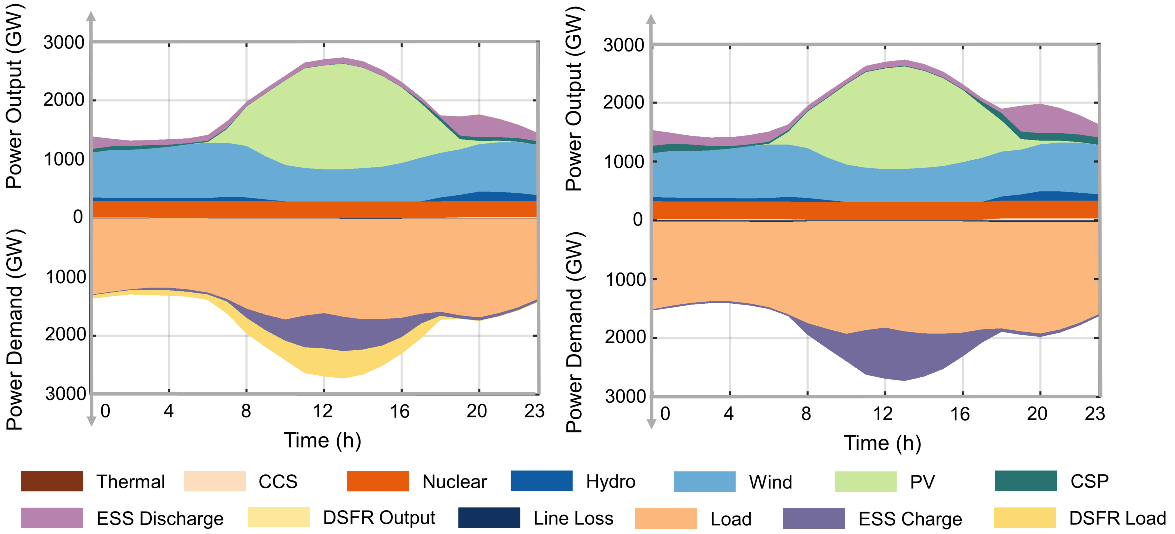

((a)) Hourly operation results of the typical day 4 in the ADF scenario and REF scenario

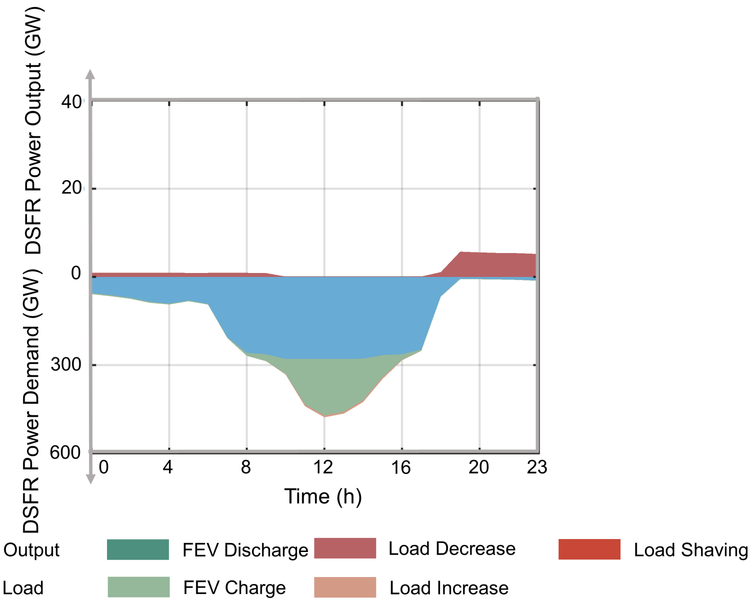

((b)) Hourly operation results of DSFR of the typical day 4 in the ADF scenario

Supplemental Figure 8. Hourly operation comparison in the typical day 4.

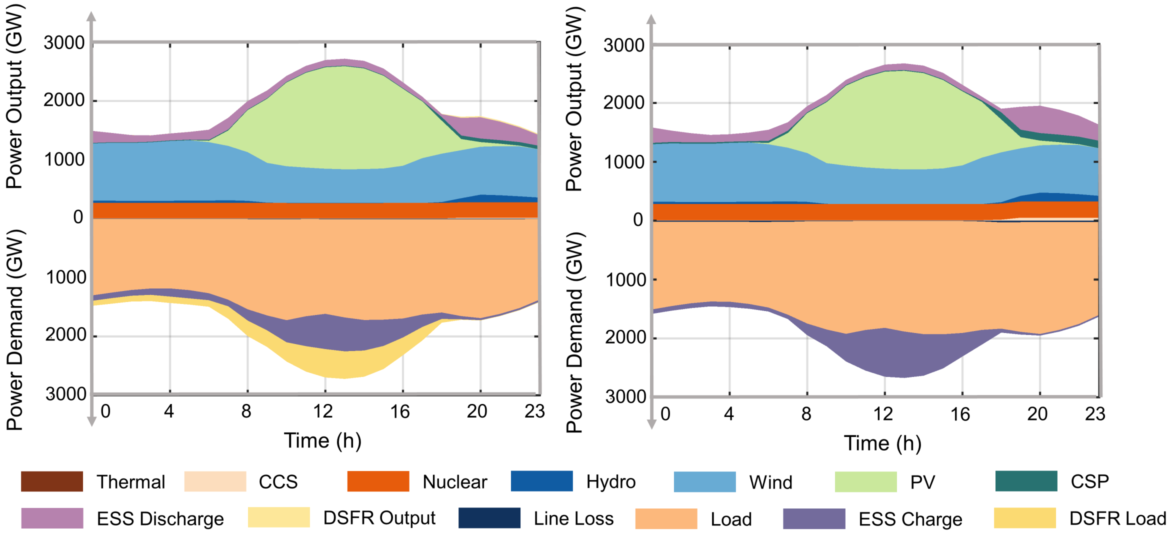

((a)) Hourly operation results of the typical day 5 in the ADF scenario and REF scenario

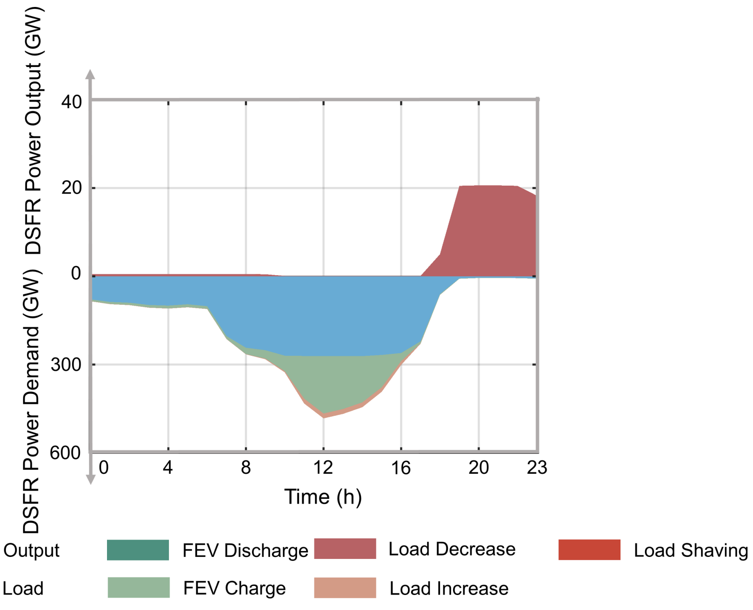

((b)) Hourly operation results of DSFR of the typical day 5 in the ADF scenario

Supplemental Figure 9. Hourly operation comparison in the typical day 5.

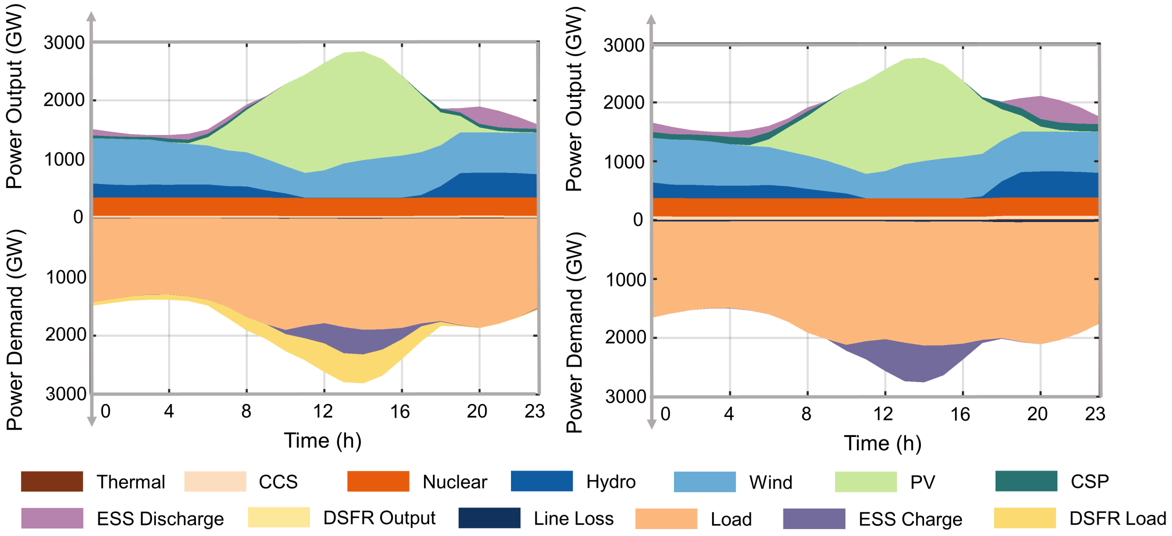

((a)) Hourly operation results of the typical day 6 in the ADF scenario and REF scenario

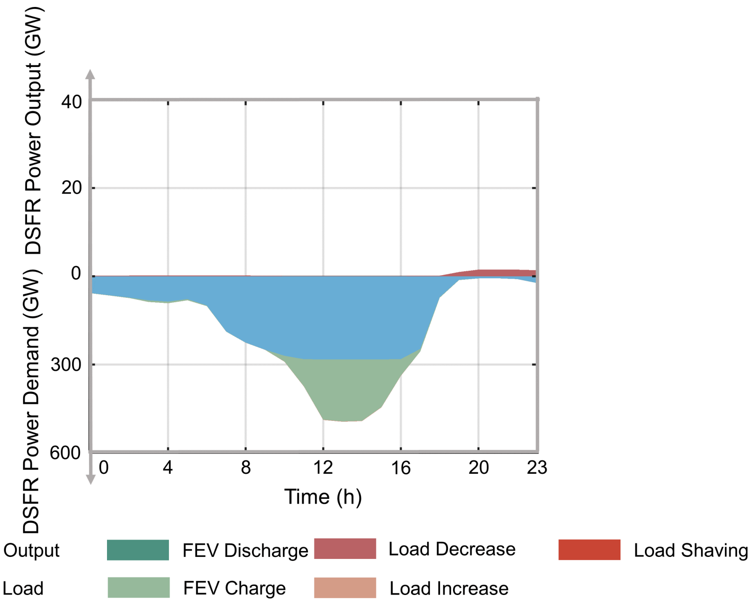

((b)) Hourly operation results of DSFR of the typical day 6 in the ADF scenario

Supplemental Figure 10. **Hourly operation comparison in the typical day 6.**

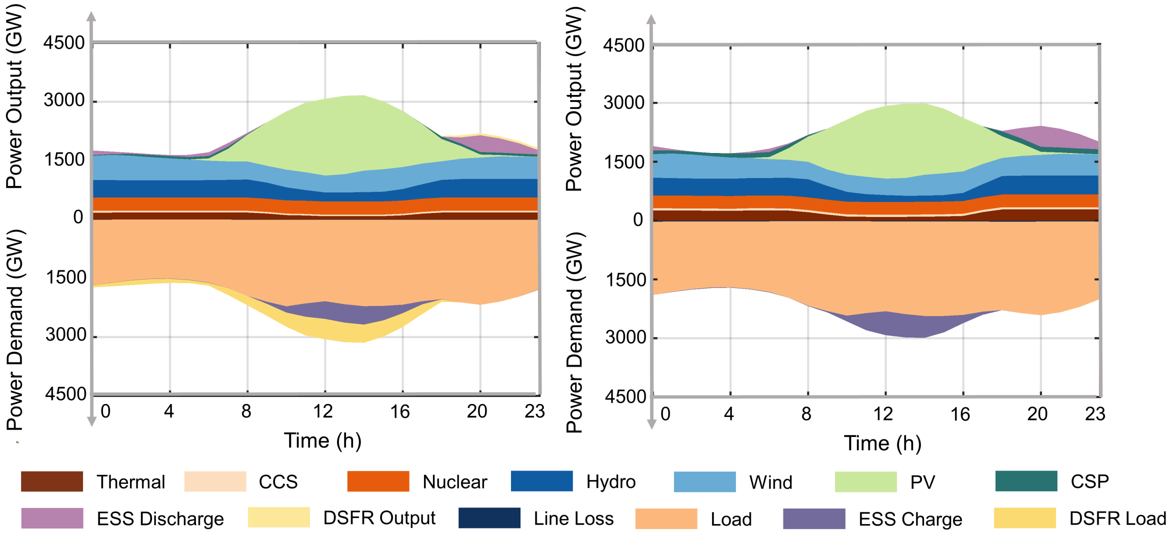

((a)) Hourly operation results of the typical day 7 in the ADF scenario and REF scenario

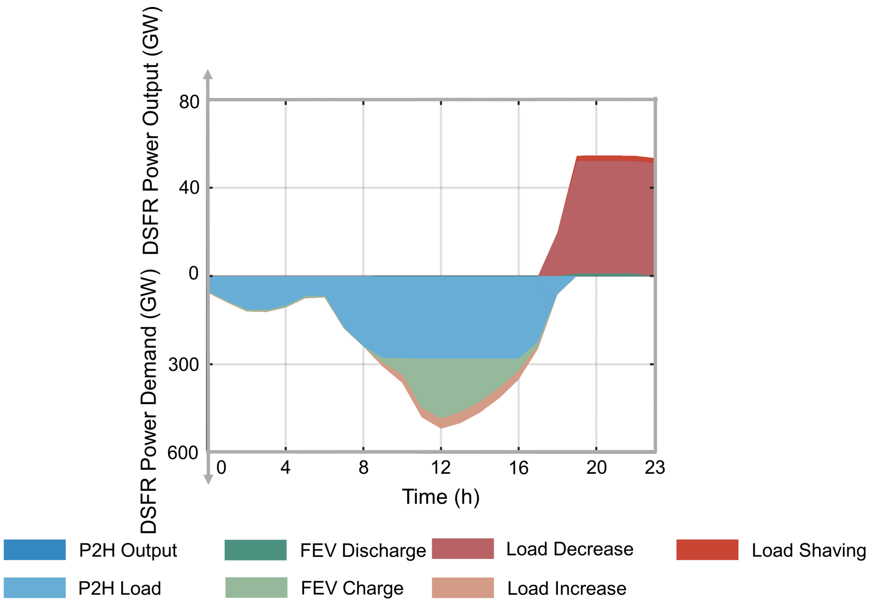

((b)) Hourly operation results of DSFR of the typical day 7 in the ADF scenario

Supplemental Figure 11. Hourly operation comparison in the typical day 7.

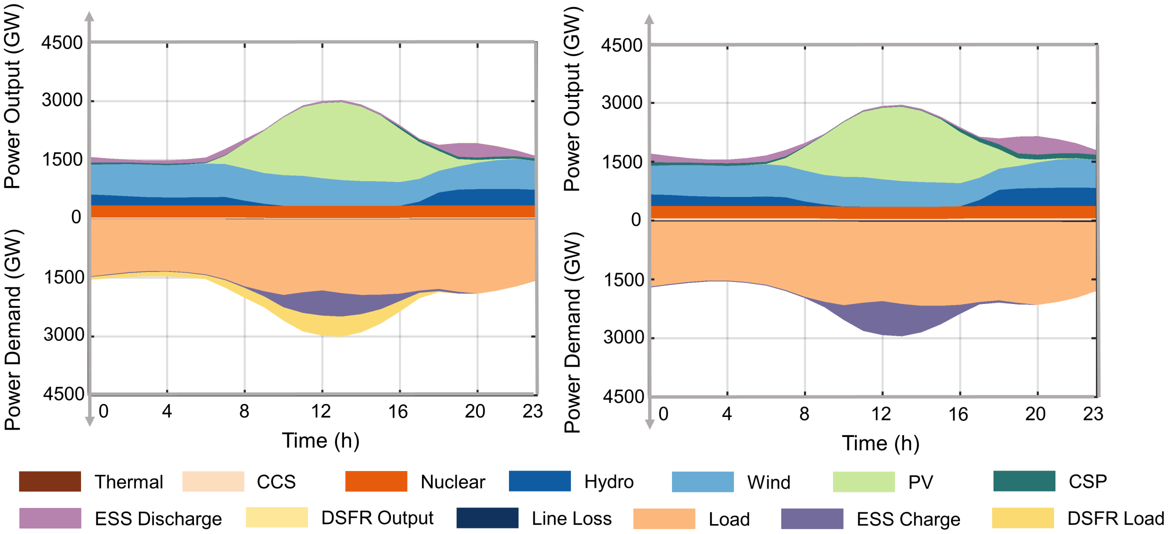

((a)) Hourly operation results of the typical day 8 in the ADF scenario and REF scenario

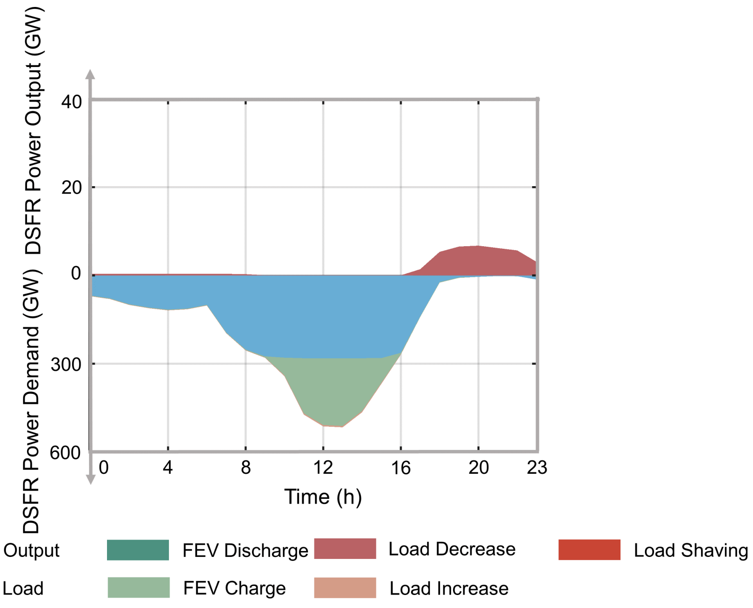

((b)) Hourly operation results of DSFR of the typical day 8 in the ADF scenario

Supplemental Figure 12. Hourly operation comparison in the typical day 8.

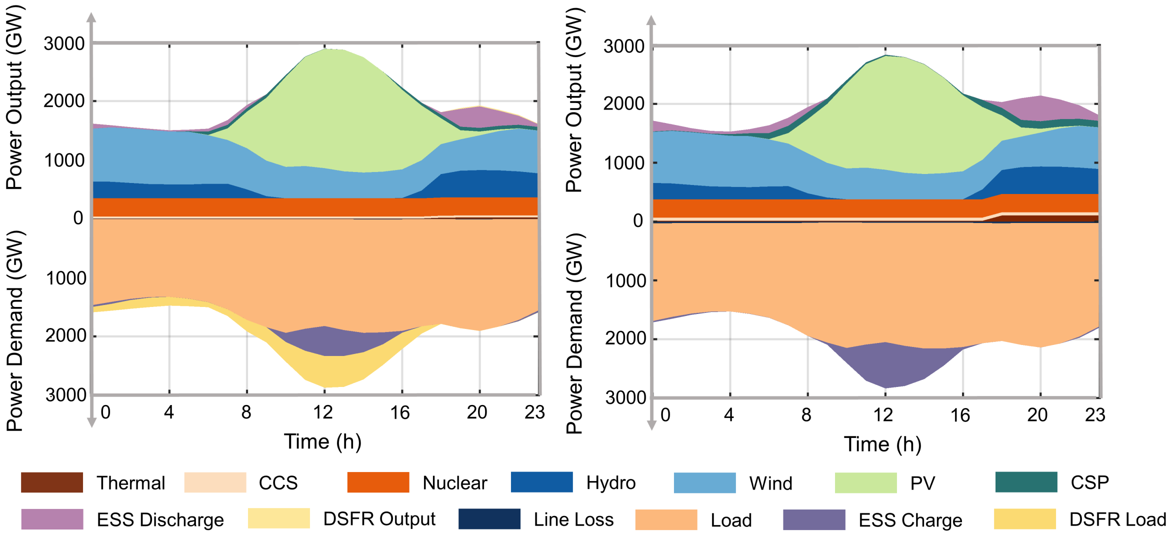

((a)) Hourly operation results of the typical day 9 in the ADF scenario and REF scenario

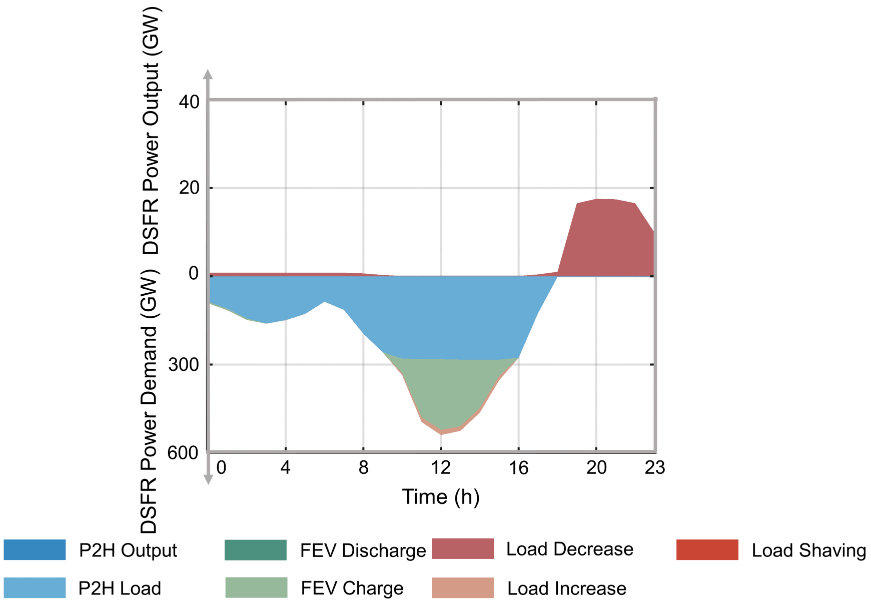

((b)) Hourly operation results of DSFR of the typical day 9 in the ADF scenario

Supplemental Figure 13. Hourly operation comparison in the typical day 9.

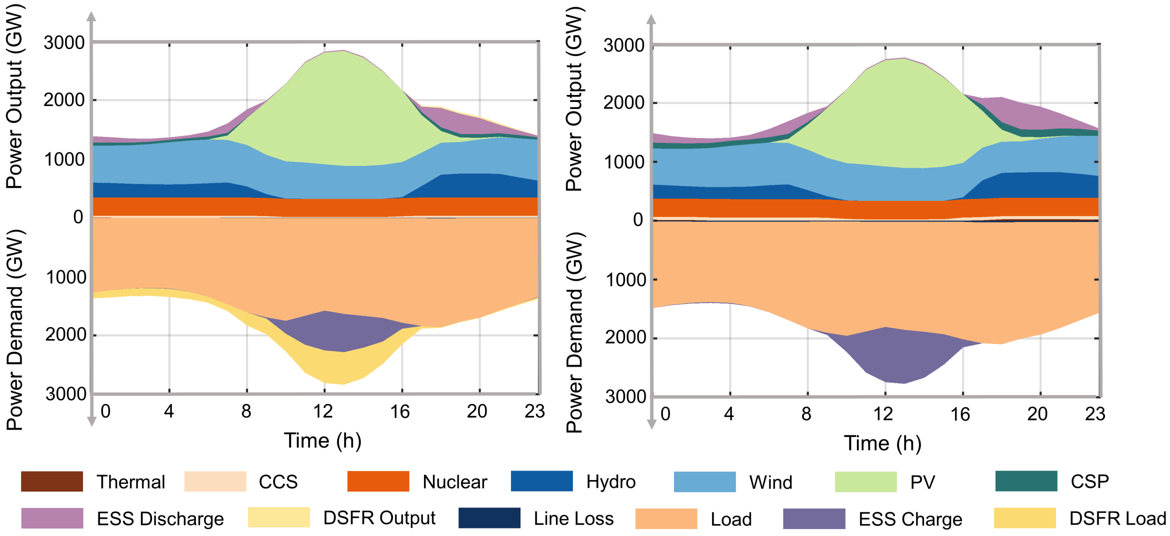

((a)) Hourly operation results of the typical day 10 in the ADF scenario and REF scenario

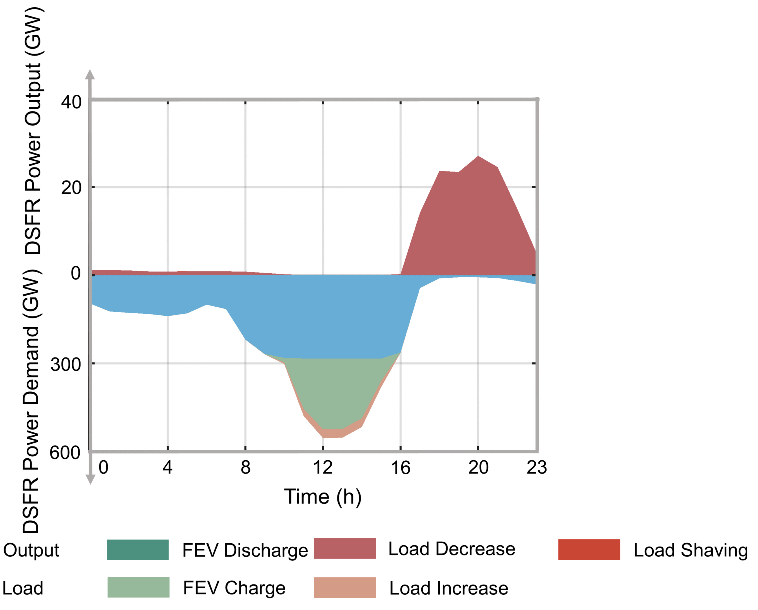

((b)) Hourly operation results of DSFR of the typical day 10 in the ADF scenario

Supplemental Figure 14. Hourly operation comparison in the typical day 10.

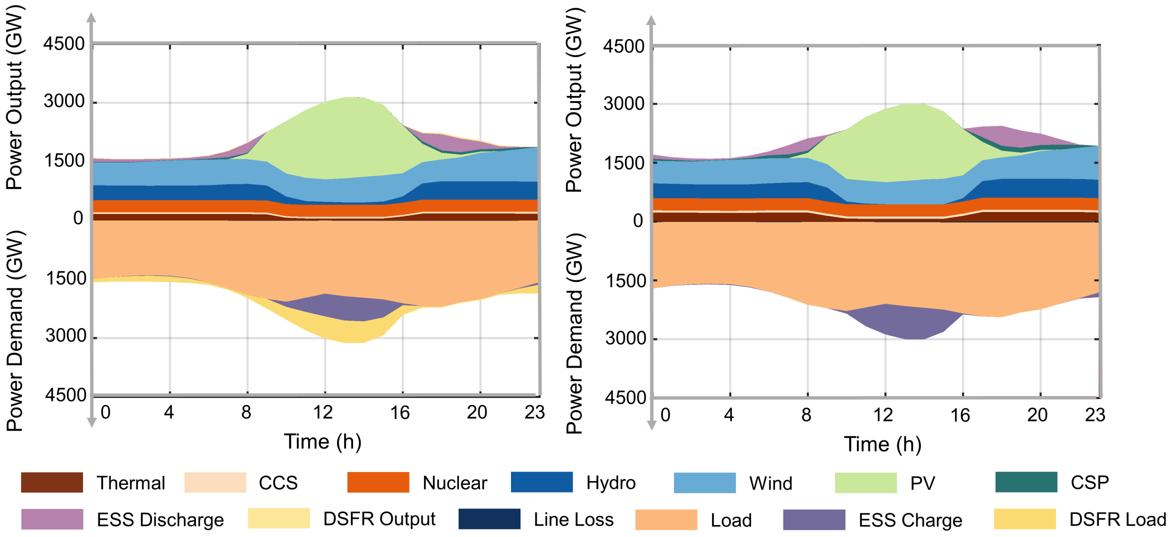

((a)) Hourly operation results of the typical day 11 in the ADF scenario and REF scenario

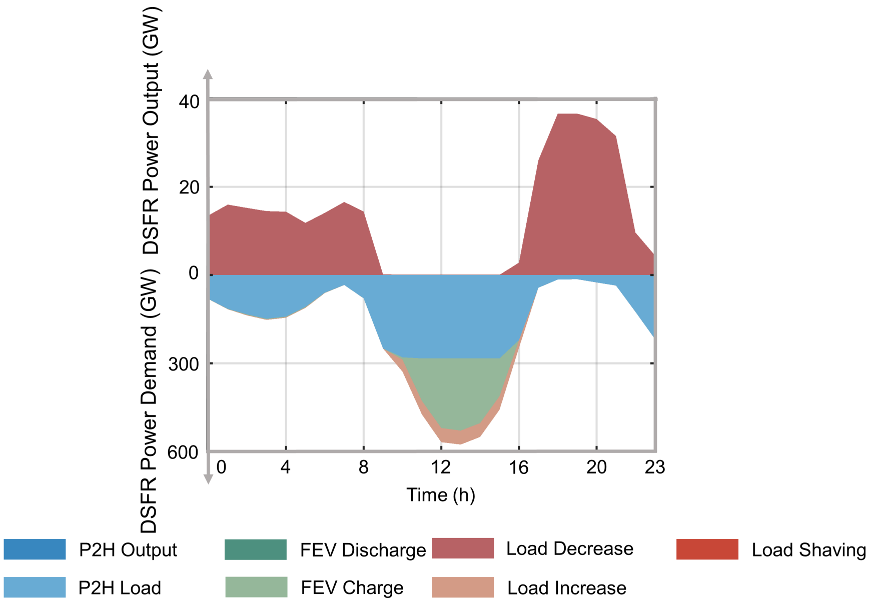

((b)) Hourly operation results of DSFR of the typical day 11 in the ADF scenario

Supplemental Figure 15. Hourly operation comparison in the typical day 11.

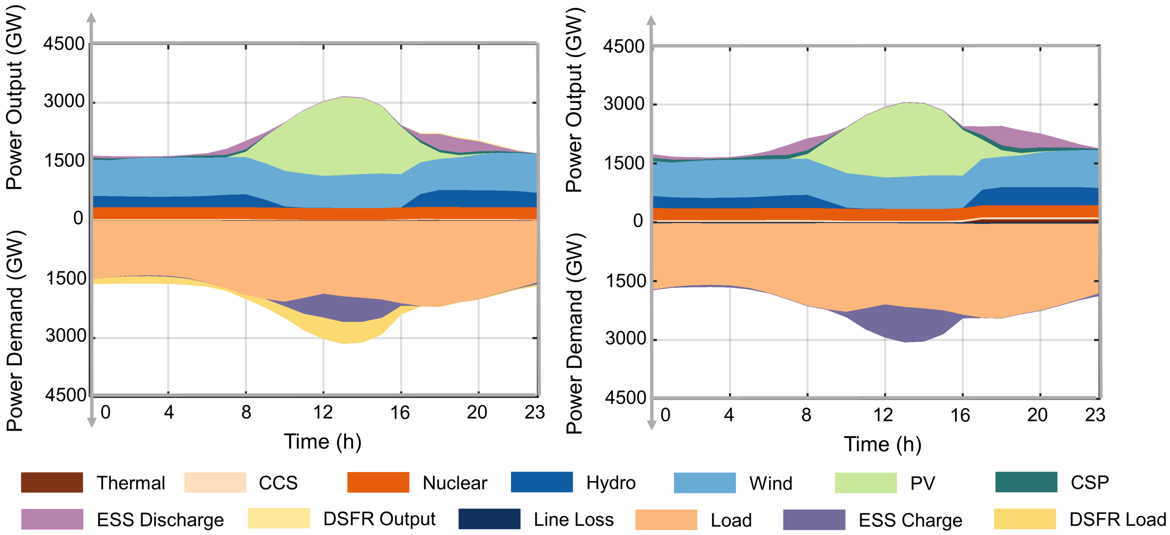

((a)) Hourly operation results of the typical day 12 in the ADF scenario and REF scenario

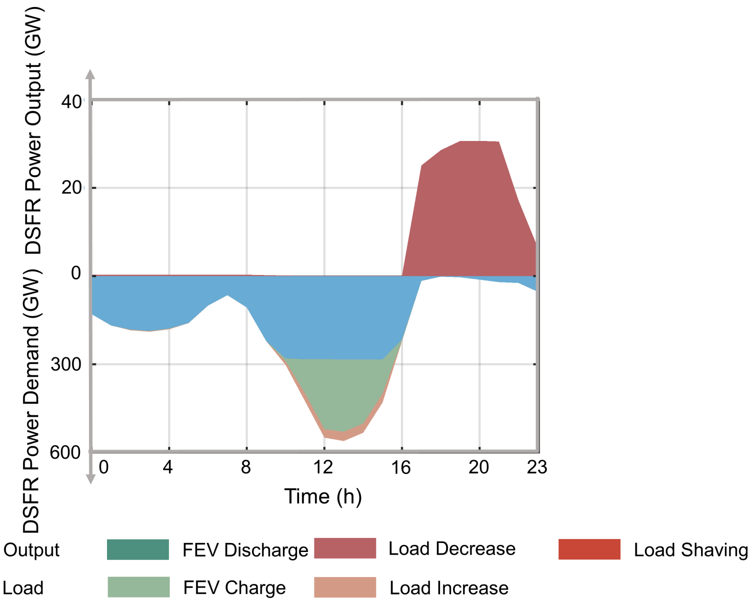

((b)) Hourly operation results of DSFR of the typical day 12 in the ADF scenario

Supplemental Figure 16. Hourly operation comparison in the typical day 12.

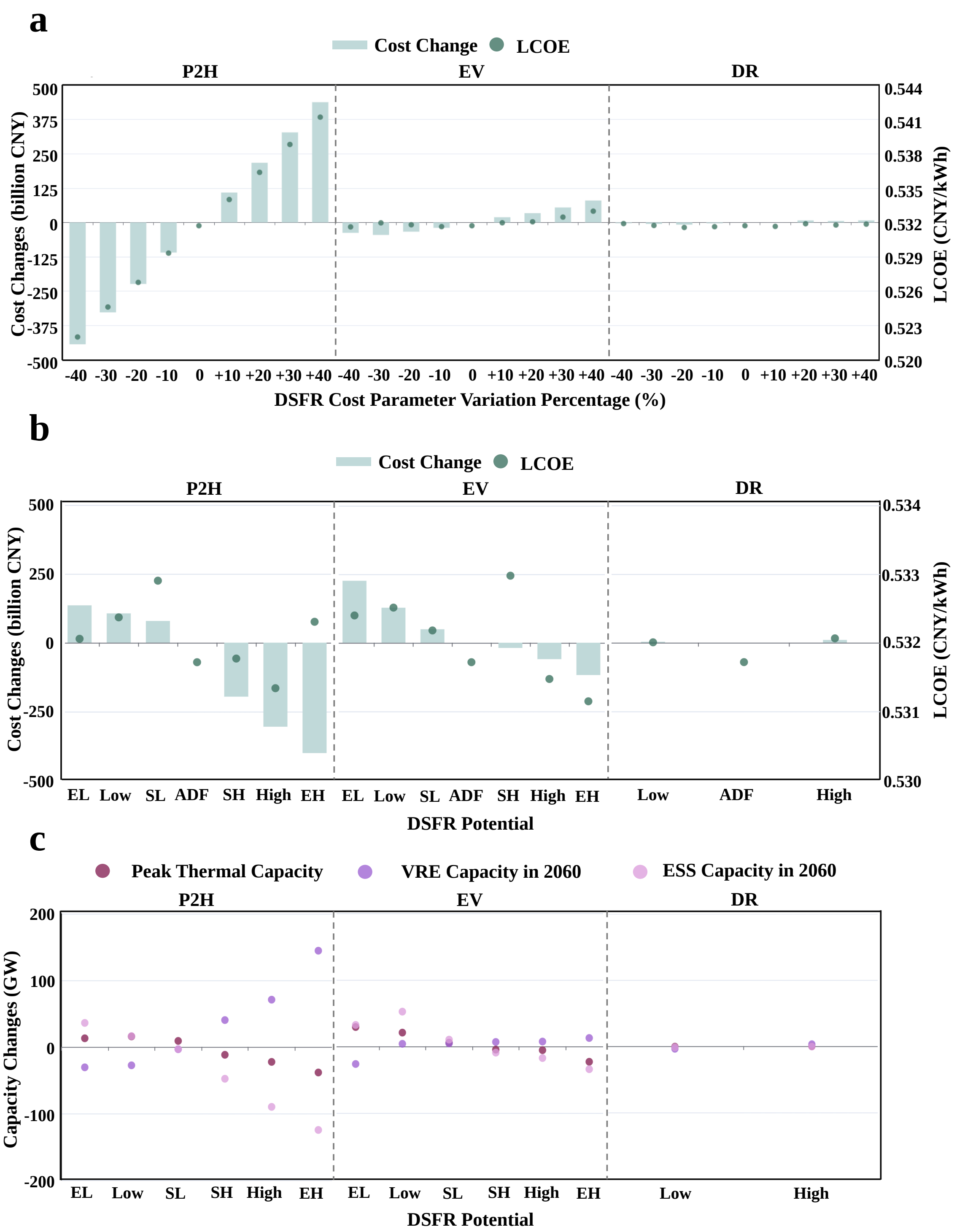

Supplemental Figure 17. Sensitivity analysis results comparison.

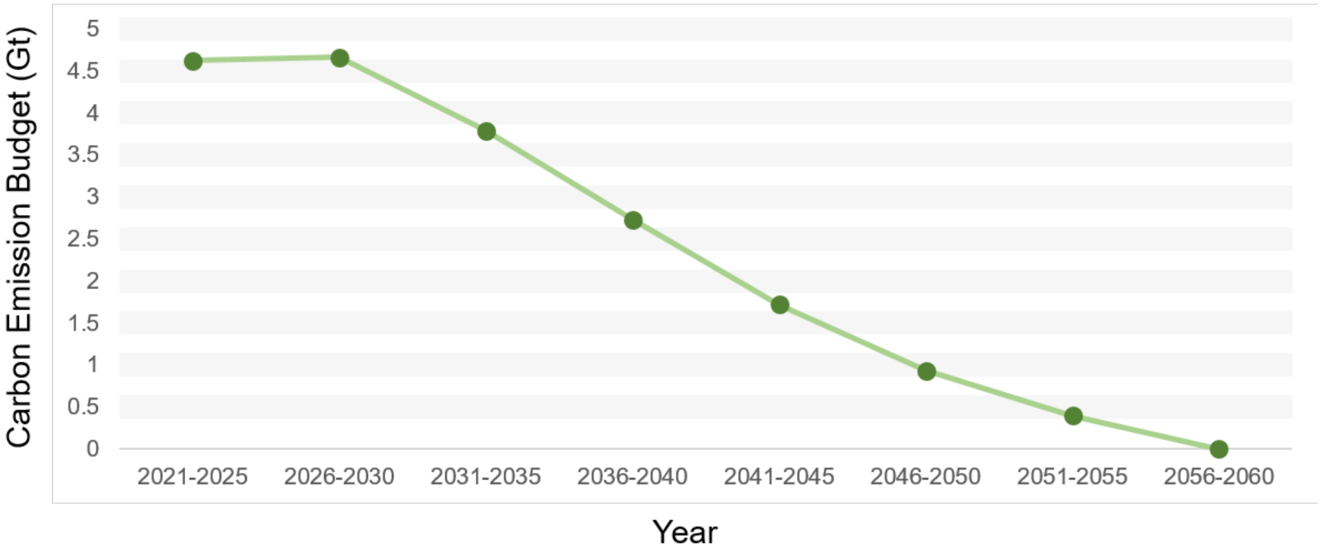

Supplemental Figure 18. **Carbon emission pathway of China's power system.**.

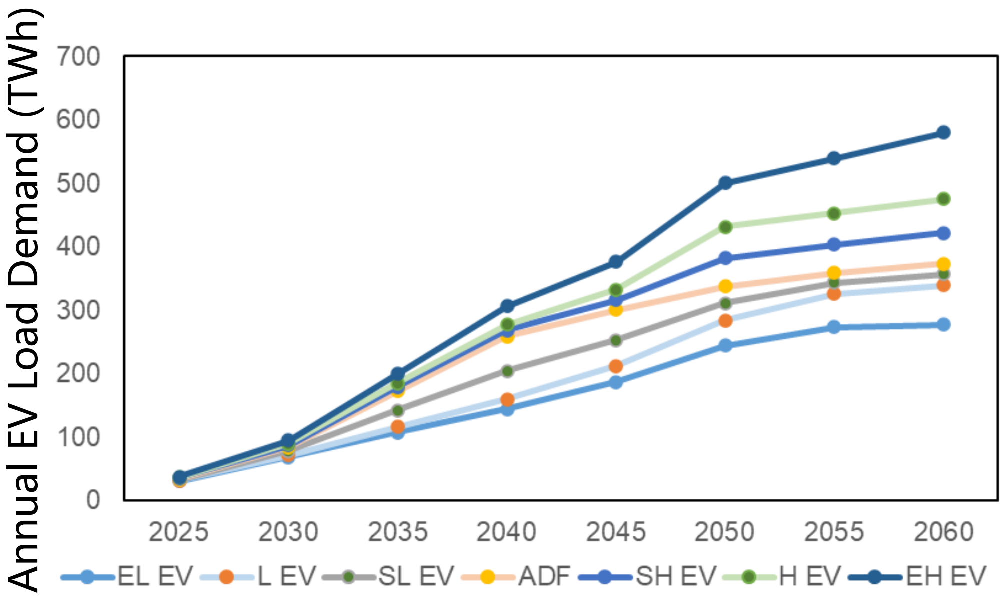

Supplemental Figure 19. **Annual EV load demands from 2025-2060 with various FEV capacity potentials.**

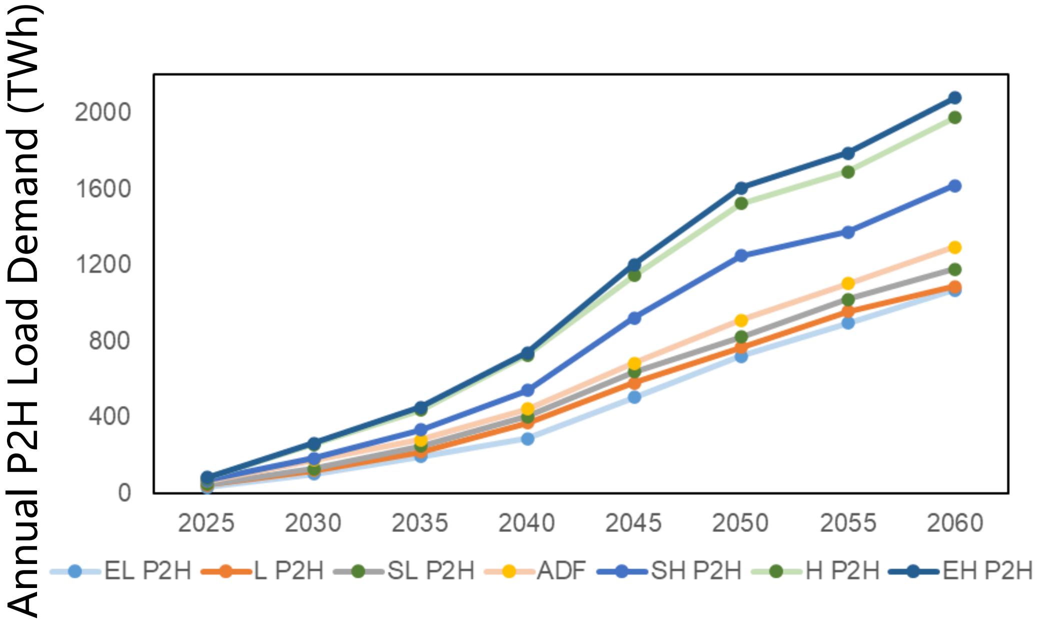

Supplemental Figure 20. **Annual P2H load demands from 2025-2060 with various P2H capacity potentials.**

Supplemental Table 1. **The annual provincial load demands from 2025 to 2060 (TWh).**

Province	2025	2030	2035	2040	2045	2050	2055	2060
Beijing	140.39	161.96	177.68	187.77	197.26	206.13	216.84	227.34
Tianjin	110.91	127.62	137.56	143.01	147.14	151.19	156.31	161.30
Hebei	451.86	510.23	540.87	540.77	530.34	520.67	521.23	521.36
Shanxi	268.03	306.45	328.59	338.44	342.45	347.53	355.90	363.96
Neimenggu	435.04	504.12	548.08	571.28	590.29	608.71	630.73	652.05
Liaoning	275.04	308.59	324.48	325.79	322.89	319.49	319.97	319.66
Jilin	88.25	99.89	105.17	105.48	106.36	106.89	109.16	111.32
Heilongjiang	116.06	132.08	140.08	142.28	145.10	147.41	151.65	155.65
Shanghai	196.96	223.51	236.63	243.63	249.65	255.12	262.52	269.37
Jiangsu	854.25	975.85	1042.73	1061.04	1079.63	1099.29	1127.77	1154.42
Zhejiang	641.21	736.86	789.39	821.74	849.94	874.92	907.64	938.66
Anhui	309.77	353.09	375.95	387.66	396.73	404.88	416.12	426.60
Fujian	306.21	350.79	375.83	390.13	401.90	413.59	427.89	441.51
Jiangxi	193.17	224.51	243.99	258.08	270.30	282.41	295.40	309.22
Shandong	776.52	896.94	970.84	1013.35	1045.52	1076.39	1113.45	1152.67
Henan	407.03	472.41	512.86	542.52	568.35	593.77	620.79	650.46
Hubei	278.65	324.74	354.22	372.15	390.59	408.77	428.02	448.72
Hunan	232.51	270.28	293.85	311.38	328.16	344.89	362.70	380.39
Guangdong	874.93	1049.87	1179.18	1244.45	1318.31	1390.28	1470.47	1550.10
Guangxi	247.27	294.49	328.69	347.63	364.03	380.28	399.14	417.78
Hainan	46.11	54.49	60.24	63.39	66.27	69.20	72.45	75.65
Chongqing	154.02	185.16	208.47	225.31	242.13	259.73	277.09	294.66
Sichuan	351.20	425.76	482.50	520.30	560.79	602.43	642.72	683.25
Guizhou	191.97	231.00	260.13	276.39	293.23	309.77	327.49	345.05
Yunnan	224.11	268.32	300.48	316.44	333.08	349.45	368.05	386.42
Xizang	13.60	16.59	18.95	20.69	22.46	24.24	25.89	27.44
Shaanxi	246.13	304.09	352.58	396.86	444.50	497.74	544.37	593.44
Gansu	165.10	205.12	239.08	271.15	305.06	343.44	377.43	413.13
Qinghai	94.41	117.39	136.91	155.41	175.06	197.22	216.83	237.41
Ningxia	136.62	167.55	193.50	216.54	239.42	266.22	290.07	315.37
Xinjiang	364.25	453.05	528.49	600.08	676.12	761.95	836.96	915.53

Supplemental Table 2. **The annual provincial EV charging load demands from 2025 to 2060 (TWh).**

Province	2025	2030	2035	2040	2045	2050	2055	2060
Beijing	2.47	5.56	6.48	7.54	8.78	10.22	10.46	10.71
Tianjin	0.59	1.39	2.67	3.11	3.18	3.26	3.33	3.41
Hebei	0.87	3.19	7.58	14.56	16.95	19.74	20.19	20.64
Shanxi	1.32	3.13	6.01	7.00	8.15	8.34	8.54	8.74
Neimenggu	0.25	0.91	2.17	4.16	4.84	5.64	6.57	7.65
Liaoning	0.41	1.50	3.57	6.86	7.99	9.31	10.84	12.62
Jilin	0.12	0.53	1.94	4.62	5.37	6.26	6.41	6.48
Heilongjiang	0.12	0.53	1.94	4.62	5.37	6.26	6.33	6.40
Shanghai	1.98	4.70	9.02	10.51	10.75	10.88	11.00	11.25
Jiangsu	2.01	4.76	11.31	26.86	31.28	32.02	32.77	33.54
Zhejiang	2.60	6.17	14.66	17.07	19.87	23.14	23.40	23.93
Anhui	0.87	3.03	7.21	8.39	8.59	8.69	8.79	8.98
Fujian	1.56	3.70	7.12	8.29	9.65	9.87	9.98	10.09
Jiangxi	0.51	1.79	4.26	4.96	5.77	6.72	7.82	8.00
Shandong	3.17	7.54	14.48	16.86	19.64	22.86	26.62	27.25
Henan	2.89	6.85	13.16	15.33	17.85	20.78	24.20	24.77
Hubei	0.51	1.88	4.46	8.58	9.99	11.63	13.54	13.86
Hunan	1.58	3.75	7.21	8.40	8.59	8.80	9.00	9.22
Guangdong	4.08	9.70	18.64	35.80	41.68	48.54	49.63	50.74
Guangxi	1.30	3.08	5.92	6.89	8.03	9.35	9.45	9.67
Hainan	0.48	1.14	2.18	2.54	2.60	2.63	2.66	2.72
Chongqing	1.05	2.48	4.77	5.55	5.69	5.75	5.82	5.95
Sichuan	1.21	2.11	5.02	9.64	11.22	13.07	15.21	17.72
Guizhou	0.34	0.81	1.93	3.70	4.31	5.02	5.08	5.19
Yunnan	0.65	1.54	3.66	7.03	8.19	9.53	9.64	9.86
Xizang	0.00	0.00	0.02	0.04	0.08	0.15	0.28	0.54
Shaanxi	0.45	0.78	1.85	3.56	4.15	4.83	5.62	6.55
Gansu	0.08	0.31	0.73	1.41	2.70	3.15	3.22	3.30
Qinghai	0.05	0.17	0.40	0.76	1.46	1.70	1.74	1.79
Ningxia	0.06	0.20	0.48	0.93	1.79	2.08	2.13	2.18
Xinjiang	0.19	0.68	1.62	3.12	5.99	6.98	8.13	9.46

Supplemental Table 3. **The annual provincial P2H load demands from 2025 to 2060 (TWh).**

Province	2025	2030	2035	2040	2045	2050	2055	2060
Beijing	0.03	0.09	0.14	0.23	0.35	0.47	0.56	0.66
Tianjin	1.17	3.15	5.08	8.06	12.41	16.51	20.02	23.50
Hebei	11.69	31.31	50.52	80.16	123.46	164.25	199.14	233.80
Shanxi	4.21	11.29	18.21	28.89	44.50	59.21	71.78	84.27
Neimenggu	3.68	9.86	15.90	25.23	38.86	51.70	62.68	73.59
Liaoning	4.39	11.75	18.96	30.09	46.34	61.66	74.75	87.76
Jilin	0.80	2.15	3.47	5.50	8.48	11.28	13.68	16.06
Heilongjiang	0.67	1.79	2.89	4.58	7.05	9.38	11.37	13.35
Shanghai	1.13	3.03	4.89	7.77	11.96	15.92	19.30	22.65
Jiangsu	5.95	15.94	25.72	40.81	62.86	83.63	101.39	119.04
Zhejiang	0.72	1.93	3.12	4.95	7.63	10.14	12.30	14.44
Anhui	1.91	5.11	8.25	13.09	20.17	26.83	32.53	38.19
Fujian	0.81	2.17	3.49	5.54	8.54	11.36	13.77	16.17
Jiangxi	1.33	3.56	5.74	9.11	14.04	18.67	22.64	26.58
Shandong	6.69	17.91	28.89	45.85	70.61	93.95	113.90	133.72
Henan	2.60	6.97	11.24	17.83	27.46	36.54	44.30	52.01
Hubei	1.62	4.34	7.01	11.12	17.12	22.78	27.62	32.43
Hunan	1.27	3.39	5.47	8.68	13.37	17.79	21.57	25.32
Guangdong	1.45	3.89	6.27	9.95	15.32	20.38	24.71	29.01
Guangxi	1.80	4.82	7.78	12.35	19.02	25.30	30.68	36.02
Hainan	0.43	1.16	1.87	2.97	4.58	6.09	7.38	8.67
Chongqing	1.02	2.74	4.41	7.00	10.79	14.35	17.40	20.43
Sichuan	1.33	3.56	5.74	9.11	14.02	18.66	22.62	26.56
Guizhou	0.32	0.85	1.37	2.18	3.35	4.46	5.40	6.34
Yunnan	1.09	2.92	4.71	7.47	11.50	15.30	18.55	21.78
Xizang	0.00	0.00	0.00	0.00	0.00	0.00	0.00	0.00
Shaanxi	2.27	6.08	9.80	15.56	23.96	31.87	38.65	45.37
Gansu	0.69	1.84	2.97	4.71	7.26	9.66	11.71	13.75
Qinghai	0.33	0.89	1.43	2.27	3.49	4.65	5.63	6.61
Ningxia	2.12	5.68	9.16	14.53	22.38	29.78	36.10	42.39
Xinjiang	1.16	3.10	5.00	7.93	12.22	16.26	19.71	23.14

Supplemental Table 4. The investment cost and operation cost parameters of DR resources.

Province	Peak-shaving DR resources investment cost (CNY/MW)	Load-shifting DR resources investment cost (CNY/MW)	Peak-shaving DR resources operation cost (CNY/MWh)	Load-shifting DR resources operation cost (CNY/MWh)
Beijing	10000	10000	2000	100
Tianjin	10000	10000	2000	100
Hebei	10000	10000	8000	100
Shanxi	10000	10000	8000	100
Neimenggu	10000	10000	8000	100
Liaoning	10000	10000	8000	100
Jilin	10000	10000	8000	100
Heilongjiang	10000	10000	8000	100
Shanghai	10000	10000	8000	100
Jiangsu	10000	10000	8000	100
Zhejiang	10000	10000	4000	100
Anhui	10000	10000	8000	100
Fujian	10000	10000	8000	100
Jiangxi	10000	10000	8000	100
Shandong	10000	10000	8000	100
Henan	10000	10000	9000	100
Hubei	10000	10000	8000	100
Hunan	10000	10000	8000	100
Guangdong	10000	10000	8000	100
Guangxi	10000	10000	8000	100
Hainan	10000	10000	8000	100
Chongqing	10000	10000	8000	100
Sichuan	10000	10000	8000	100
Guizhou	10000	10000	8000	100
Yunnan	10000	10000	8000	100
Xizang	10000	10000	8000	100
Shaanxi	10000	10000	10000	100
Gansu	10000	10000	10000	100
Qinghai	10000	10000	8000	100
Ningxia	10000	10000	2000	100
Xinjiang	10000	10000	8000	100

[1]These cost parameters are set according to policies in different provinces[1–16].

References

1. People's Government of Shanghai City. Shanghai city carbon peaking implementation plan. https://www.ndrc.gov.cn/fggz/hjyzy/tdftzh/202208/t20220808_1332758.html (2022).
2. People's Government of Guangdong Province. Guangdong province carbon peaking implementation plan. http://www.gd.gov.cn/zwgk/gongbao/2023/4/content/post_4091315.html (2022).
3. Ningxia Development and Reform Commission. Power demand response regulations in ningxia hui autonomous region. https://fzggw.nx.gov.cn/tzgg/202206/t20220614_3559903.html?eqid=febf498b000c81c8000000056437f705 (2022).
4. Shandong Development and Reform Commission, Shandong Energy Bureau. Provincial interruptible power load demand response work plan in 2022. http://nyj.shandong.gov.cn/art/2022/6/7/art_59960_10292617.html (2022).
5. Fujian Development and Reform Commission. Power demand response work plan in fujian province (trial). http://fgw.fj.gov.cn/zfxxgkzl/zfxxgkml/bwgfxwj/202205/t20220524_5916577.htm (2022).
6. Chongqing Economic and Information Technology Commission. Chongqing grid demand response work plan (trial) (2022).
7. Hebei Development and Reform Commission. Hebei province power demand response market operation rules. http://info.hebei.gov.cn/hbszfxxgk/329975/329988/330035/6852718/7016112/index.html (2022).
8. Guizhou Energy Bureau. Guizhou province power demand response work plan (trial). http://www.guizhou.gov.cn/zwgk/zdlygk/jjgzlfz/nyzy/dlgl/202307/t20230711_80838290.html (2022).
9. Anhui Energy Bureau. Anhui province power demand response work plan (trial). https://fzggw.ah.gov.cn/group6/M00/06/E1/wKg8BmOQCmKAbc6UAAcl-uZkj-Q858.pdf?eqid=bc39658a0001253a00000006648a8aef (2022).
10. Commisson of Industry and Information Technology of Guangxi Zhuang Autonomous Region. Guangxi power demand response market work plan (draft) (2022).
11. Tianjin Industrial and Information Technology Bureau. Tianjin power demand response detailed regulations in 2022. https://gyxxh.tj.gov.cn/ZWGK4147/ZCWJ6355/wjwj/202201/t20220124_5787811.html (2022).
12. Zhejiang Development and Reform Commission, Zhejiang Energy Bureau. 2021 power demand response work implementation notice. https://fzggw.zj.gov.cn/art/2021/6/8/art_1229629046_4906648.html (2021).
13. Shaanxi Development and Reform Commission. Shaanxi province power demand response work plan in 2021. http://sndrc.shaanxi.gov.cn/fgwj/2021nwj/MVFvEj.htm (2021).
14. Shaanxi Development and Reform Commission. Jiangsu power demand response detailed regulations (revised draft for comments). http://fzggw.jiangsu.gov.cn/art/2022/10/24/art_284_10637935.html (2022).
15. Henan Development and Reform Commission. Notice on ensuring power supply. https://fgw.henan.gov.cn/2022/05-23/2454193.html (2022).
16. Inner Mongolia Energy Bureau. Mengxi grid demand-side response detailed regulations in inner mongolia autonomous region (version 1.0). http://nyj.nmg.gov.cn/zwgk/zfxxgkzl/fdzdgknr/tzgg_16482/tz_16483/202209/t20220905_2123928.html (2022).
